# Polyanionic C-Terminal IDR-Dependent Phase Separation of DrHsp17 Contributes to Acid Stress Protection in *Deinococcus radiodurans*

**DOI:** 10.3390/ijms27219801

**Published:** 2026-10-31

**Authors:** Ning Yu, Shang Dai, Binqiang Wang, Chunhui Cai, Liangyan Wang, Yuejin Hua, Bing Tian

**Affiliations:** 1 Institute of Biophysics, College of Life Sciences, Zhejiang University, Hangzhou 310058, China; 11916016@zju.edu.cn (N.Y.); wangbinqiang@zju.edu.cn (B.W.); 12107139@zju.edu.cn (C.C.); liangyanwang@zju.edu.cn (L.W.); yjhua@zju.edu.cn (Y.H.); 2 College of Life Sciences, Nanjing Agricultural University, Nanjing 210095, China; daishang@njau.edu.cn; 3 Zhejiang University-Lishui Joint Innovation Center for Life and Health & Lishui Lvgu Institute for Life and Health, Lishui 323010, China

**Keywords:** *Deinococcus radiodurans*, DrHsp17, small heat shock protein, acid stress, phase separation

## Abstract

The extremophilic bacterium *Deinococcus radiodurans* is a promising chassis for bioremediating acidic radioactive environments, yet how it protects intracellular proteins against acid stress remains poorly understood. Here, we investigate the atypical small heat shock protein DrHsp17 to elucidate its physiological role and biophysical properties under acid stress. Our results show that DrHsp17 contributes to cell survival under severe acidic conditions, which induce liquid–liquid phase separation (LLPS) of DrHsp17 to form dynamic condensate droplets both in vivo and in vitro. The phase-separation behavior of DrHsp17 depends on its polyanionic C-terminal intrinsically disordered region (IDR). Notably, LLPS is associated with enhanced chaperone activity of DrHsp17 and reduced intracellular protein aggregation. These findings indicate polyanionic IDR-driven phase separation of the DrHsp17 as a novel mechanism involved in bacterial acid stress-sensing and resistance, with the potential for engineering *D*. *radiodurans* cells for bioremediation under acid stress.

## 1. Introduction

Acidic environments—prevalent in the acidic soils, industrial effluents, and radioactive contamination sites, even in the mammalian gastrointestinal tract—impose severe stresses on the survival of bacterial cells [1,2,3]. For most bacteria, exposure to low pH not only disrupts intracellular physicochemical homeostasis and membrane potential, but also induces extensive protein unfolding, misfolding, and irreversible aggregation, ultimately leading to lethal proteotoxicity [2,3]. To counteract such conditions, bacteria have evolved diverse defensive strategies, including energy-coupled proton extrusion [4], amino acid decarboxylation-dependent metabolic buffering [5,6], envelope remodeling [7], and the deployment of acid-activated molecular chaperones, such as the periplasmic chaperone HdeA [8]. Among these stress responses, chaperone-mediated maintenance of protein homeostasis (proteostasis) constitutes a critical frontline defense for cellular survival under extreme acid stress [1,3,8].

The extremophilic bacterium *Deinococcus radiodurans*, renowned for its extraordinary resistance to ionizing radiation, oxidative stress, and desiccation [9,10], has emerged as a promising biological chassis for the bioremediation of heavy metal– and radionuclide-contaminated acidic wastes, particularly radioactive uranium-bearing wastewaters [11,12,13,14]. The highly toxic soluble U(VI) mainly exists in wastewater in the form of free uranyl ions under acidic conditions (pH  <  5) [15]. However, the rapid advancement of engineering applications of *D*. *radiodurans* in radioactive acidic wastewater outpaces the fundamental understanding of its acid tolerance mechanisms [14,16]. Direct evidence concerning the acid stress response in *D. radiodurans* remains scarce, except for observations of acid-induced DNA damage and identification of acid uracil-DNA glycosylase such as DR0022 [17,18]. Compared to its extensively characterized DNA repair machinery [9,10], it remains largely unknown how *D. radiodurans* protects its intracellular proteins against damage when confronted with severe environmental acidification.

Small heat shock proteins (sHsps) act as ATP-independent molecular chaperones involved in the cellular protein quality control network, rapidly binding non-native folding intermediates to prevent their irreversible aggregation under various stresses [19,20]. To exert this protective function and facilitate downstream refolding cascades by multichaperone networks, the majority of bacterial sHsps assemble into large, dynamic oligomeric complexes [19,21,22]. However, prior structural and biochemical analyses have revealed that DrHsp17 (also designated as Hsp17.7), one of the two sHsps encoded by *D. radiodurans*, exhibits highly atypical characteristics [23]. Unlike canonical sHsps, DrHsp17 exists predominantly as an elongated dimer and interacts with unfolding substrates only transiently and weakly under conventional heat stress [23]. These features raise the question of how DrHsp17 is reorganized and functionally engaged under severe acid stress, because the canonical model of stress-induced remodeling of higher-order sHsp oligomers may not readily apply to a protein that is predominantly dimeric under basal conditions.

A growing body of evidence has established liquid–liquid phase separation (LLPS) as an important mechanism by which cells rapidly reorganize biomolecules in response to environmental stress [24,25]. Intracellular pH changes can act as potent biophysical regulators of phase behavior by altering electrostatic interactions and the multivalent interaction potential of intrinsically disordered regions (IDRs) [26,27,28]. Stress-responsive LLPS has been extensively characterized in eukaryotic cells, where condensates often reorganize RNA metabolism, translation, signaling, and protein quality control during environmental stress [24,25,29]. In bacteria, phase separation has increasingly been implicated in diverse cellular processes, including genome organization, RNA metabolism, cell division, signaling, metabolic regulation, and stress adaptation [30,31,32]. However, the mechanisms by which bacterial small heat shock proteins may couple pH-responsive phase separation to chaperone-mediated proteostasis, particularly through defined intrinsically disordered regions, remain incompletely understood [33].

In this study, we aimed to elucidate the physiological role of DrHsp17 in *D. radiodurans* during acid stress and to investigate whether phase separation contributes to its stress-responsive chaperone function. Specifically, we examined (i) whether DrHsp17 contributes to survival under severe acid stress, (ii) whether DrHsp17 undergoes pH-dependent condensation in vivo and in vitro, (iii) which structural features of DrHsp17 are required for this phase behavior, and (iv) how disruption of these features affects its protein-protective function. Through these analyses, we sought to clarify how an atypical, predominantly dimeric bacterial sHsp may be reorganized under severe acid stress.

## 2. Results

### 2.1. DrHsp17 Contributes to the Acid Stress Response of Deinococcus radiodurans

To investigate the role of DrHsp17 in acid stress tolerance of *D. radiodurans*, we constructed a *drhsp17* (GenBank accession: ANC71207.1) deletion mutant (*Δdrhsp17*) via homologous recombination, replacing the target gene with a kanamycin resistance cassette (Figure S1A). The successful deletion was verified by PCR using locus-specific primers (Figure S1B). The *Δdrhsp17* mutant, along with the wild-type (WT) and a *drhsp17* overexpression strain (*+drhsp17*), was then used to evaluate cell viability under various stress conditions.

**Figure 1:**
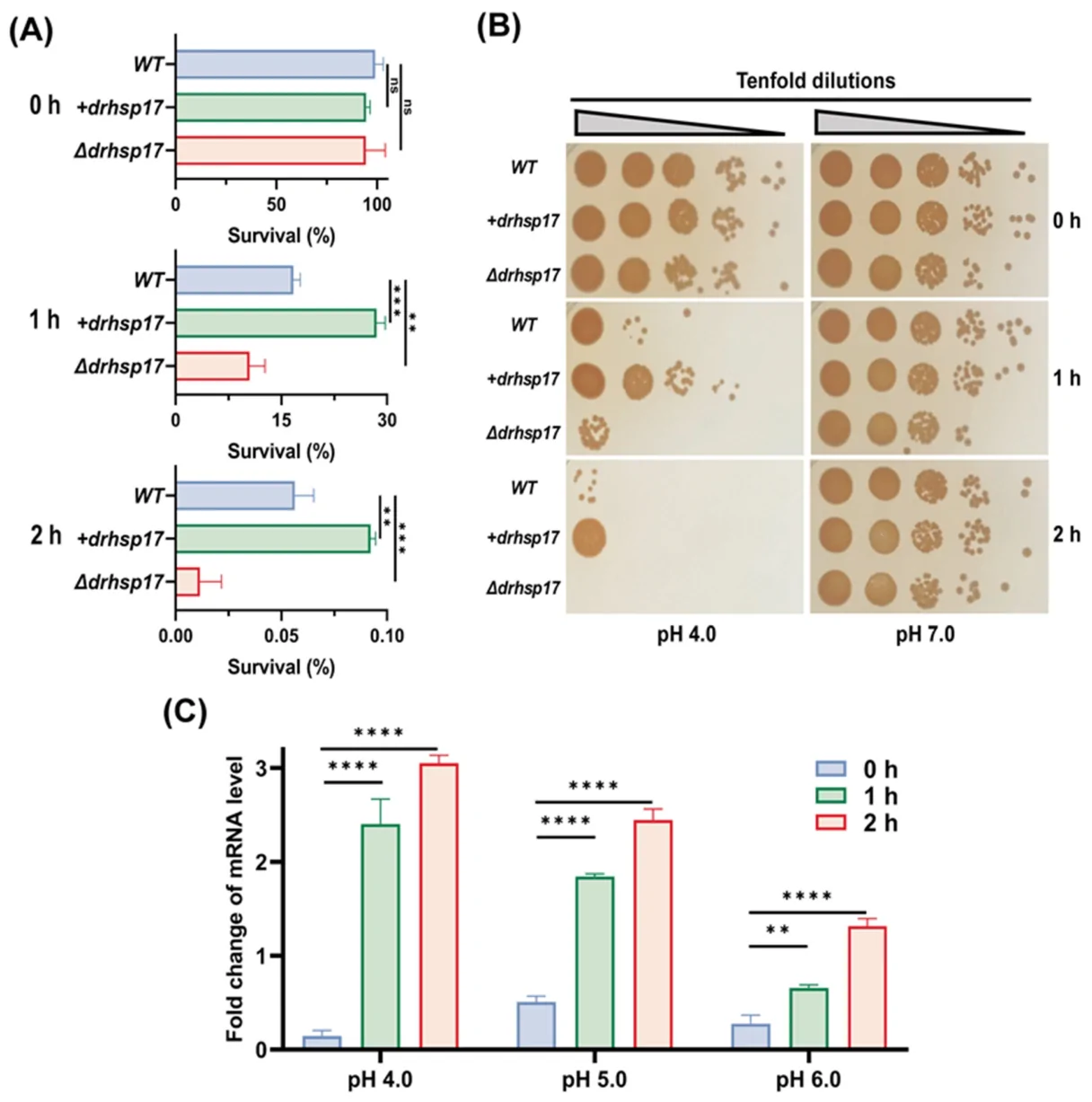
DrHsp17 contributes to the acid stress response of *D*. *radiodurans*. (**A**) Survival fractions (%) of the wild-type (WT), *drhsp17* overexpression (*+drhsp17*), and *drhsp17* deletion (*Δdrhsp17*) strains after exposure to pH 4.0 acid stress for 0, 1, and 2 h. (**B**) Survival phenotypic assays of the WT, *+drhsp17*, and *Δdrhsp17* strains under acid stress (pH 4.0). Cells were treated with pH stress buffer (40 mM Na_2_HPO_4_, 20 mM citric acid, 137 mM NaCl, 2.7 mM KCl) at pH 4.0 or pH 7.0 control for 0, 1, and 2 h, respectively. Tenfold serial dilutions of the cultures following acid stress treatments were spotted onto agar plates. (**C**) Relative mRNA expression levels of *drhsp17* in the WT strain under different acid stress. Cells were exposed to pH 4.0, 5.0, or 6.0 for 0, 1, and 2 h, respectively, and transcript levels were determined by RT-qPCR. Data in (**A**,**C**) are presented as mean ± SD of three independent biological replicates. Statistical significance was determined by one-way ANOVA followed by Dunnett’s multiple-comparisons test, performed separately for each pH condition, with the 0 h group serving as the control. **, *p* < 0.01; ***, *p* < 0.001; ****, *p* < 0.0001; ns, not significant.

Under neutral (pH 7.0), mildly acidic (pH 6.0), and mildly alkaline (pH 8.0) conditions, all three strains exhibited comparable colony growth without obvious differences (Figure 1A,B, Figures S2 and S3A), indicating that *drhsp17* is dispensable for basal growth and survival under neutral or mild pH fluctuations. Under severe acid stress (pH 4.0), the spot assay revealed only a modest difference in growth/survival between the *Δdrhsp17* mutant and the wild-type strain, whereas the *drhsp17* overexpression strain showed somewhat improved resistance (Figure 1B). Quantitative survival analysis, however, detected a statistically significant reduction in the survival fraction of the *Δdrhsp17* mutant and an increased survival fraction of the *+drhsp17* strain relative to the wild type after acid exposure (Figure 1A). These results indicate that DrHsp17 contributes to, but is not indispensable for, survival of *D. radiodurans* under severe acid stress. We further examined the transcriptional response of *drhsp17* to varying pH levels using RT-qPCR. In the wild-type strain, *drhsp17* transcripts were significantly upregulated under all tested acidic conditions (pH 4.0, 5.0, and 6.0) at 1 h or 2 h relative to the control without acid stress (0 h) (Figure 1C and Figure S4). The induction was pH-dependent, with the strongest response induced at pH 4.0. A slight upregulation of *drhsp17* was also observed under mild alkaline stress (pH 8.0) (Figure S3B), while the survival phenotype of *Δdrhsp17* was similar to the wild type at mildly alkaline (pH 8.0) (Figure S3A), indicating that the transcriptional induction of *drhsp17* might be decoupling from survival phenotypic necessity.

We also tested cell viability under several other stress conditions and whether the decoupling between transcriptional induction and phenotypic necessity applies to general stress responses such as oxidative (H_2_O_2_), ultraviolet (UV) radiation, and heat treatments (Figure S5). Similar to the response to pH fluctuations, *drhsp17* transcription was slightly upregulated upon exposure to H_2_O_2_ and heat stress, and downregulated under UV radiation (Figure S5D–F). However, despite these transcriptional changes, the Δ*drhsp17* and the +*drhsp17* strains exhibited no obvious survival defects or enhancements compared to the WT across all tested doses of oxidative, UV, and heat stresses (Figure S5A–C). Collectively, these expression profiles and phenotypic assays indicate that while *drhsp17* transcription is broadly responsive to various environmental stimuli, its protein product appears to play a particularly important role in survival under severe acid stress.

### 2.2. DrHsp17 Harbors an Intrinsically Disordered and Highly Negatively Charged C-Terminal Region

**Figure 2:**
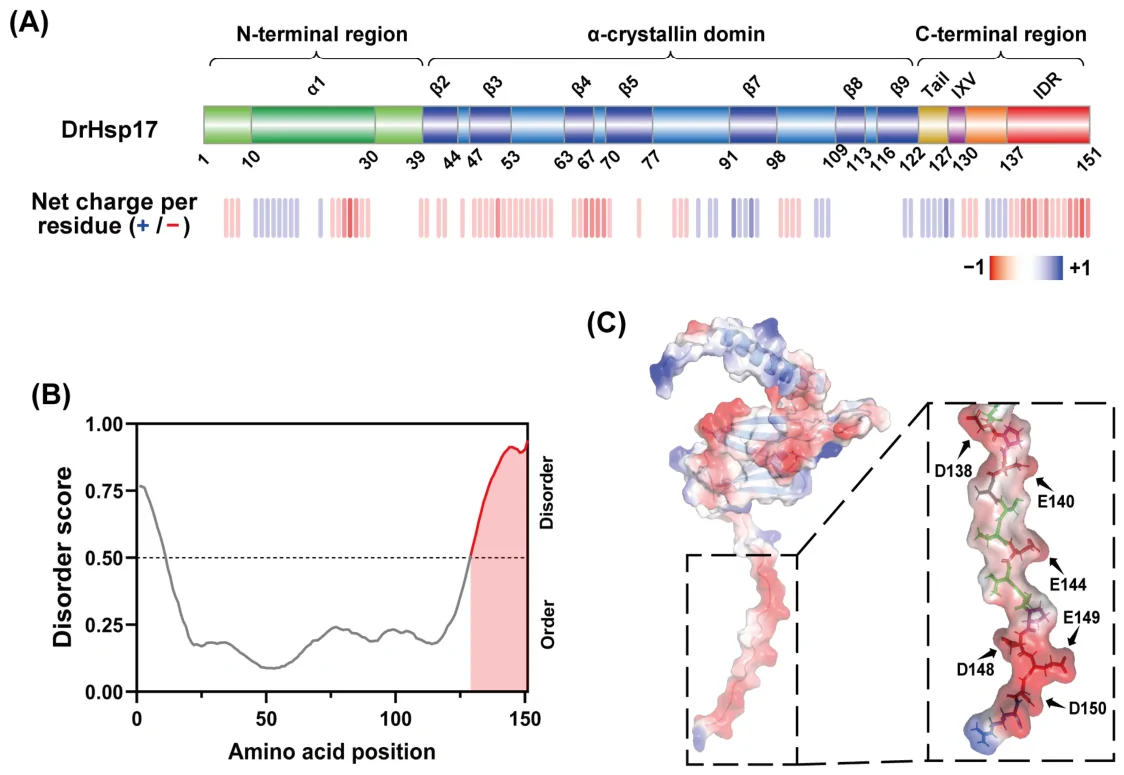
Sequence analysis and IDR prediction reveal an intrinsically disordered and negatively charged region in the C-terminal of DrHsp17. (**A**) Schematic of DrHsp17 domain architecture (upper) and corresponding NCPR profile (lower). Red and blue bars represent negatively and positively charged residue calculations, respectively. (**B**) Prediction of IDR in DrHsp17 using IUPred3 long-disorder prediction. The disorder score is plotted against the amino acid position. The region with scores exceeding 0.5 (red background) corresponds to the predicted C-terminal IDR. (**C**) Three-dimensional structural model of DrHsp17 predicted by AlphaFold, with the molecular surface colored by electrostatic potential (red, negative; blue, positive; white, neutral). The magnified view highlights the C-terminal IDR enriched in anionic residues. The anionic Asp (D) and Glu (E) residues are indicated by arrows.

To elucidate the structural basis underlying the role of DrHsp17 in acid tolerance, we performed comprehensive sequence and structural bioinformatics analyses of DrHsp17. First, we analyzed the domain architecture of DrHsp17 (Figure 2A). The sequence analysis illustrates that DrHsp17 consists of an N-terminal region containing an α1 helix, a conserved central α-crystallin domain (β2–β9), and a C-terminal region containing an IXV motif followed by a predicted IDR. The net charge per residue (NCPR) calculation of DrHsp17 using the CIDER tool (https://pappulab.wustl.edu/CIDER/analysis/, accessed on 16 February 2026) shows a highly asymmetric charge distribution. The C-terminal IDR is heavily populated with negatively charged amino acids, compared with the relatively balanced charge distribution in the rest of the protein (Figure 2A). Although another region with negative charge density is observed between residues ~40 and 60, this sequence constitutes part of the highly structured α-crystallin core domain with a very low disorder score (Figure 2B) and is distinct from the phase-separation-driving IDR.

Next, we evaluated the conformational flexibility of the C-terminal IDR of DrHsp17 using the IUPred3 server (https://iupred3.elte.hu/, accessed on 14 August 2025). As shown in Figure 2B, while the central core domain and more than 70% of the N-terminal region exhibit low disorder scores (below 0.5), the C-terminal region (residues 122–151) shows a sharp increase in disorder scores. This indicates the presence of an intrinsically disordered region (IDR) at the C-terminus. The AlphaFold model of DrHsp17 (AlphaFold Database entry: AF-Q9RTR5-F1) predicts the central α-crystallin domain with substantially higher confidence than the C-terminal region (Figure 2C), whereas the C-terminal segment is assigned low prediction confidence. We therefore do not interpret the predicted model as structural confirmation of this region; rather, the low-confidence prediction is consistent with the IUPred3-based assignment of the C-terminus as intrinsically disordered. This interpretation is also consistent with the available crystal structure of DrHsp17 (PDB: 4FEI), in which the C-terminal segment is not resolved. Mapping the electrostatic surface potential onto the predicted model reveals a pronounced electronegative surface (red) at the disordered C-terminus. The magnified view highlights that this negative charge density arises from a high proportion of anionic residues—specifically aspartic acid (D) and glutamic acid (E), including D138, E140, E144, D148, E149, and D150. Notably, sequence alignment reveals that this extended polyanionic region is not conserved among other typical bacterial sHsps (Figure S6). Together, the results reveal that DrHsp17 possesses an atypical polyanionic disordered C-terminal IDR and the anionic residues may serve as key elements for sensing protons during acid stress.

### 2.3. Acid Stress-Induced Liquid–Liquid Phase Separation of DrHsp17 In Vivo and In Vitro

**Figure 3:**
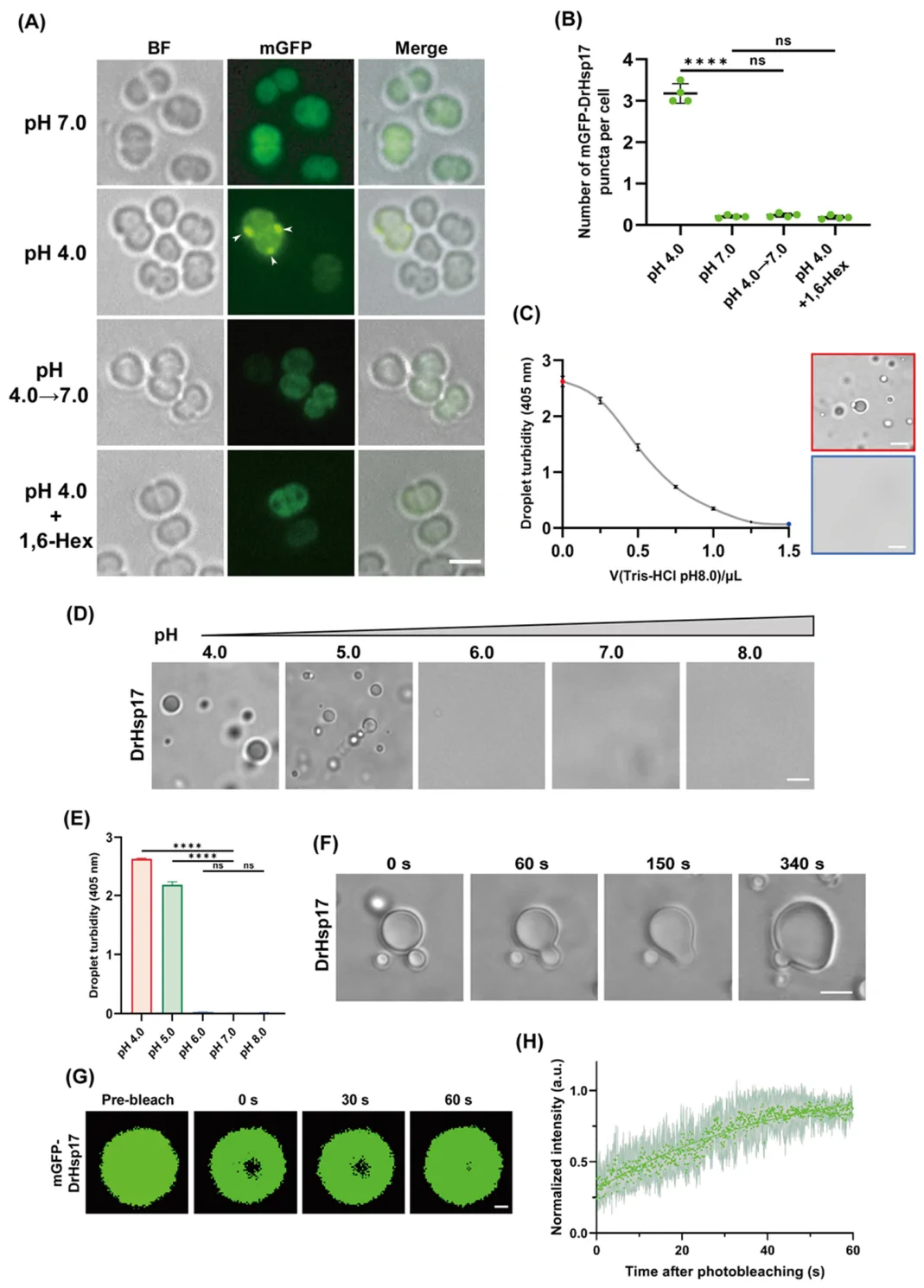
Reversible acid-induced liquid–liquid phase separation of DrHsp17 in vivo and in vitro. (**A**) Fluorescence microscopy images of *D. radiodurans* cells expressing mGFP-DrHsp17. Cells were treated with LLPS buffer (20 mM Na_2_HPO_4_, 20 mM citric acid, 150 mM KCl) at pH 7.0 or pH 4.0 for 1 h. For the recovery experiment, cells exposed to pH 4.0 were subsequently returned to pH 7.0 conditions (pH 4.0 → 7.0). For the dissolution assay, 12.5% 1,6-hexanediol (1,6-Hex) was applied. BF, bright field. Scale bar, 0.5 μm. BF, bright field. (**B**) Quantification of the number of mGFP-DrHsp17 fluorescent puncta per cell under the same conditions as in (**A**). Each data point represents the number of puncta per cell quantified from an individual 30 × 30 µm field of view. Data are presented as mean ± SD from four independent biological experiments. Statistical significance was determined by one-way ANOVA followed by Dunnett’s multiple-comparisons test. ****, *p* < 0.0001; ns, not significant. (**C**) Reversibility of DrHsp17 droplet formation in vitro upon increasing pH. Purified DrHsp17 (20 μM, 200 μL) was first induced to form droplets under acidic conditions, followed by stepwise addition of 100 mM Tris-HCl (pH 8.0). Droplet turbidity was monitored at 405 nm as increasing volumes of Tris-HCl were added. Data are mean ± SD of three independent replicates. Representative microscopy images before titration (red) and at the titration endpoint after droplet dissolution (blue) are shown on the right. Scale bar: 5 μm. (**D**) Representative microscopy images of pH-dependent phase separation of purified DrHsp17 in vitro. 20 μM protein was incubated in LLPS buffer at pH 4.0 to 8.0. Scale bar, 5 μm. (**E**) Turbidity (OD_405_) of DrHsp17 solutions (20 μM) under the pH conditions as shown in (**D**). Data are mean ± SD of three independent replicates. Statistical significance was determined by one-way ANOVA followed by Dunnett’s multiple-comparisons test. ****, *p* < 0.0001; ns, not significant. (**F**) Time-lapse imaging of droplet fusion. The assay was performed with 30 μM purified DrHsp17 at pH 4.0. Scale bar, 5 μm. (**G**) FRAP assay of mGFP-DrHsp17 droplets in vitro (30 μM, pH 4.0). Representative images show the droplet before bleaching (Pre-bleach), immediately after photobleaching (0 s), and post-bleach (30 s and 60 s). Scale bar: 0.2 μm. (**H**) Normalized fluorescence recovery curve of mGFP-DrHsp17 droplets in vitro. Data are mean ± SD of three independent replicates.

To investigate the spatiotemporal response of DrHsp17 to acid stress, we examined the subcellular distribution of mGFP-DrHsp17 in *D. radiodurans*. Under neutral conditions (pH 7.0), mGFP-DrHsp17 was predominantly distributed throughout the cytoplasm, whereas exposure to pH 4.0 for 1 h induced the formation of distinct fluorescent puncta (Figure 3A). Quantitative analysis confirmed a marked increase in the number of mGFP-DrHsp17 puncta per cell under pH 4.0 compared with pH 7.0 (Figure 3B). Importantly, when acid-treated cells were returned to pH 7.0 conditions, the puncta largely disappeared and the puncta number decreased to near-basal levels, demonstrating that the acid-induced intracellular condensation of DrHsp17 is reversible (Figure 3A,B). Treatment with 12.5% 1,6-hexanediol similarly reduced puncta formation under acidic conditions. Because 1,6-hexanediol is not a specific probe for LLPS, we interpret this chemical perturbation as supportive evidence, whereas the pH-recovery experiment provides an independent demonstration of reversibility. This reversibility indicates that DrHsp17 forms dynamic biomolecular condensates in vivo via LLPS. Further dead cell staining using Ethidium Homodimer III (EthD-III) verified that the dynamic puncta are physiological structures in viable cells rather than autofluorescent artifacts of dying cells (Figure S7).

To determine whether phase separation is an intrinsic property of DrHsp17, we purified recombinant wild-type DrHsp17 and mGFP-DrHsp17 (Figure S8) for in vitro reconstitution. We next examined whether the acid-induced phase transition was also reversible in vitro. Purified DrHsp17 (20 μM) was first induced to form droplets under acidic conditions, after which increasing amounts of 100 mM Tris-HCl (pH 8.0) were added to progressively increase the pH. The turbidity at 405 nm decreased continuously during titration and approached the baseline at the endpoint, accompanied by complete disappearance of visible droplets by microscopy (Figure 3C). Thus, DrHsp17 droplets can be reversibly dissolved by increasing the pH, indicating that the acid-induced condensates represent a reversible phase transition rather than irreversible acid-induced protein precipitation. Consistent with in vivo observations, the DrHsp17 exhibited pH-dependent phase separation behavior. Microscopic imaging (Figure 3D) and turbidity measurements at 405 nm (Figure 3E) showed that DrHsp17 (20 μM) formed abundant liquid droplets under acidic conditions (pH 4.0 and 5.0), whereas the protein remained uniformly distributed in solution at pH 6.0 and above. Furthermore, the DrHsp17 LLPS in vitro was also protein concentration-dependent; droplet formation was initiated at 10 μM of DrHsp17 and droplets increased in size at higher concentrations (Figure S9A,D). Experiments with mGFP-DrHsp17 exhibited that the mGFP tag did not interfere with the intrinsic LLPS capacity of DrHsp17 (Figure S9B). Similar to the in vivo assays, in vitro DrHsp17 droplets could be progressively dissolved with the increased concentration of external 1,6-hexanediol (Figure S9C).

We next characterized the physical properties of the DrHsp17 condensates to confirm their liquid nature. Time-lapse microscopy captured the fusion events of DrHsp17 condensates, where two adjacent droplets coalesced into a single, larger spherical droplet over 340 s (Figure 3F). Additionally, we performed fluorescence recovery after photobleaching (FRAP) on the mGFP-DrHsp17 droplets in vitro. After targeted photobleaching, the fluorescence intensity within the bleached region recovered rapidly and substantially within 60 s (Figure 3G,H), indicating the internal molecular mobility in the DrHsp17 condensate. Thus, the droplet fusion events and the high internal molecular mobility confirmed that acid stress-induced DrHsp17 LLPS forms highly dynamic, liquid condensate droplets.

### 2.4. The Negatively Charged C-Terminal IDR Is Required for Acid-Induced Phase Separation of DrHsp17

**Figure 4:**
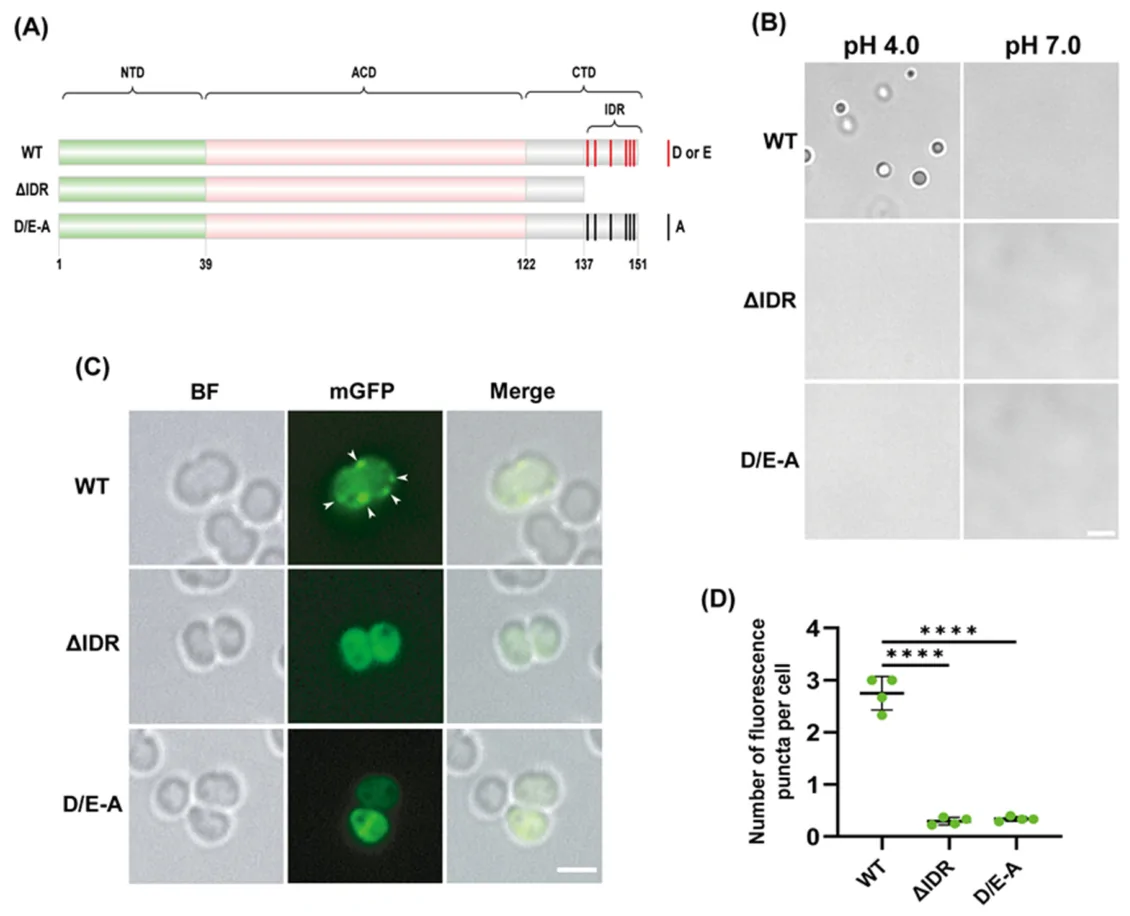
The C-terminal IDR and its negatively charged residues are required for the acid-induced LLPS of DrHsp17. (**A**) Schematic illustration of the wild-type DrHsp17 and its variant proteins, including an IDR-truncated mutant (ΔIDR) and charge-altered site mutants (D/E-A). The red vertical lines indicate the six native negatively charged residues within the C-terminal IDR (D138, E140, E144, D148, E149, and D150). In the D/E-A mutant, all six residues were substituted with alanine (A, indicated by black lines). NTD, N-terminal domain. ACD, α-crystallin domain. CTD, C-terminal domain. (**B**) Representative in vitro microscopy images evaluating the phase separation capacity of the DrHsp17 and its variants. 20 μM proteins were incubated in LLPS buffer (20 mM Na_2_HPO_4_, 20 mM citric acid, 150 mM KCl) at pH 4.0 or pH 7.0. Scale bar, 5 μm. (**C**) Fluorescence microscopy images of the in vivo subcellular distribution of mGFP-fused DrHsp17 and its variants. Cells were exposed to acid stress (pH 4.0) for 1 h. White arrowheads indicate the distinct fluorescent puncta. BF, bright field. Scale bar, 0.5 μm. (**D**) Quantification of the number of mGFP-DrHsp17 fluorescent puncta per cell under the same condition as in (**C**). Each data point represents the number of puncta per cell quantified from an individual 30 × 30 µm field of view. Data are presented as mean ± SD from four independent biological experiments. Statistical significance was determined by one-way ANOVA followed by Dunnett’s multiple-comparisons test. ****, *p* < 0.0001.

Guided by our sequence analysis revealing an intrinsically disordered and highly electronegative region, we hypothesized that this region functions as the primary determinant for DrHsp17 LLPS. To test this, we generated two DrHsp17 variants (Figure 4A): a truncation mutant lacking the entire predicted IDR (ΔIDR) and a charge-neutralized mutant in which six acidic residues within the C-terminal IDR (D138, E140, E144, D148, E149, and D150) were substituted with alanine (D/E-A). Following protein purification (Figure S8), we evaluated their phase separation capacity in vitro. While the wild-type DrHsp17 readily assembled into liquid droplets in the pH 4.0 buffer, all the mutant proteins lost the ability to undergo phase separation under the identical conditions (Figure 4B). These results demonstrate that the C-terminal IDR and its negatively charged residues are required for the acid stress-induced LLPS of DrHsp17.

We next investigated whether these molecular requirements also influence DrHsp17 condensation in vivo by expressing mGFP-fused wild-type DrHsp17, ΔIDR, and D/E-A in *D. radiodurans*. Upon exposure to acid stress (pH 4.0, 1 h), wild-type DrHsp17 formed distinct intracellular fluorescent puncta, whereas both ΔIDR and D/E-A remained predominantly diffuse in the cytoplasm (Figure 4C). Quantification of the number of fluorescent puncta per cell confirmed a marked reduction in puncta formation in both mutant strains compared with wild-type DrHsp17 (Figure 4D). These in vivo observations are consistent with the in vitro findings (Figure 4B). Taken together, these results suggest that the polyanionic IDR is critical for DrHsp17 phase separation in response to acid stress.

To determine whether the loss of phase separation in the DrHsp17 variants was accompanied by alterations in soluble protein assembly, we further characterized wild-type DrHsp17, D/E-A, and ΔIDR by Native PAGE and size-exclusion chromatography (SEC) (Figure S10). Under pH 7.0 and pH 4.0 conditions, with 6% 1,6-hexanediol included under acidic conditions to disrupt condensates, each protein exhibited a predominant soluble species by Native PAGE (Figure S10B). SEC further showed that the elution profiles of wild-type DrHsp17 and D/E-A were highly similar between pH 7.0 and pH 4.0 conditions (Figure S10C,D), indicating no detectable acid-induced transition in their soluble assembly states under these conditions. The theoretical molecular weights of the purified recombinant monomeric DrHsp17, D/E-A, and ΔIDR proteins, including their N-terminal 6× His tags, calculated using ExPASy ProtParam (https://web.expasy.org/protparam/, accessed on 30 September 2025), are 18.494, 18.188, and 17.079 kDa, respectively. To facilitate interpretation of the SEC profiles, ovalbumin (~45 kDa) and lysozyme (~14.7 kDa) were analyzed as molecular weight standards under the same elution buffer as those used for the pH 7.0 samples (Figure S10F). The elution volumes of all three proteins were closer to that of ovalbumin than to that of lysozyme, suggesting that their apparent hydrodynamic sizes are more consistent with larger assemblies than with monomeric species of their theoretical molecular weights. Notably, ΔIDR eluted at a later volume than wild-type DrHsp17 (Figure S10E), potentially reflecting its reduced molecular mass and altered hydrodynamic dimensions following deletion of the C-terminal IDR. Nevertheless, the similar SEC profiles observed under pH 7.0 and pH 4.0 conditions argue against a major acid-induced rearrangement of the soluble assembly states of these proteins under the tested conditions.

### 2.5. The C-Terminal IDR and Its Associated Phase-Separation Capacity Contribute to the Protective Function of DrHsp17

**Figure 5:**
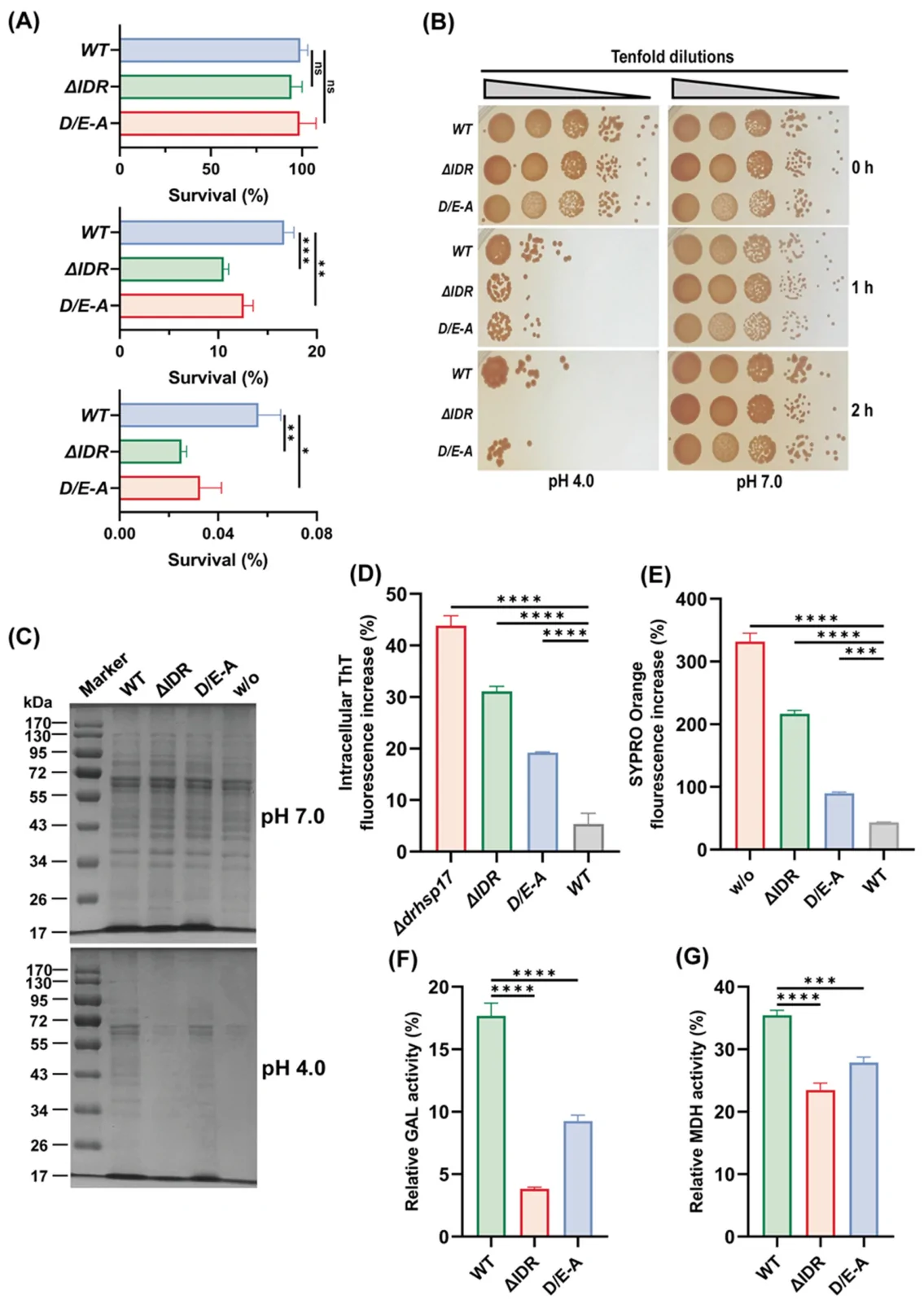
The C-terminal IDR and phase-separation capacity of DrHsp17 contribute to protein protection under acid stress. (**A**) Survival fractions (%) of the *Δdrhsp17* strains complemented with wild-type *drhsp17*, *drhsp17-ΔIDR*, or *drhsp17-D/E-A* after exposure to pH 4.0 acid stress for 0, 1, and 2 h (from top to bottom panel). (**B**) Survival phenotypic assays of *D. radiodurans* wild-type (WT) and DrHsp17 variants (*ΔIDR*, *D/E-A*). Cells were exposed to pH 4.0 or pH 7.0 for 0, 1, and 2 h before being spotted onto agar plates. (**C**) SDS-PAGE analysis of soluble proteins in *Δdrhsp17* cell extracts following acid treatment in the absence (w/o) or presence of wild-type DrHsp17 or its variants. (**D**) Intracellular protein aggregation measured by Thioflavin T (ThT) fluorescence. The relative increase in ThT fluorescence for each strain under pH 4.0 stress was calculated relative to its control under pH 7.0. (**E**) In vitro protein unfolding assay using SYPRO Orange. The protein extract of the *Δdrhsp17* strain was incubated without (w/o) or with the wild-type DrHsp17 and its variants. The relative increase in SYPRO Orange fluorescence for each sample under pH 4.0 stress was calculated relative to its control under pH 7.0. (**F**,**G**) Functional protection of the model client enzymes β-galactosidase (GAL) and malate dehydrogenase (MDH). Before measurement, 5 μL of 1 M Tris-HCl (pH 8.0) was added to dissolve condensates. Relative retained activity was calculated as (activity at pH 4.0/activity of the corresponding sample at pH 7.0) × 100%. Data in (**A**,**D**–**G**) represent the mean ± SD of three independent replicates. Statistical significance was determined using one-way ANOVA followed by Dunnett’s multiple-comparisons test. *, *p* < 0.05; **, *p* < 0.01; ***, *p* < 0.001; ****, *p* < 0.0001, ns, not significant.

Having established the acid-induced LLPS of DrHsp17, we sought to determine whether this phase transition is linked to its biological function as a molecular chaperone. We evaluated the survival of *D. radiodurans* strains expressing the phase-separation-deficient mutants under acid stress (pH 4.0). Compared with the wild-type strain, both the *ΔIDR* and *D/E-A* variants, which were deficient in phase separation, showed reduced protection against acid stress in vivo (Figure 5A,B). These results demonstrate a close association between the structural determinants required for LLPS and the protective function of DrHsp17, although the contribution of LLPS cannot be fully separated from possible direct effects of the IDR mutations themselves.

To investigate whether the reduced acid resistance was associated with increased intracellular protein aggregation, we measured protein aggregation using a Thioflavin T (ThT) fluorescence assay. Upon pH 4.0 treatment, the *Δdrhsp17* strain showed a marked increase in intracellular protein aggregation, which was significantly suppressed by the complementation of wild-type DrHsp17 (Figure 5D). However, the *ΔIDR*, *D/E-A* mutants failed to recover the protection of DrHsp17 on intracellular proteins, resulting in significantly higher intracellular protein aggregation levels than the wild-type DrHsp17 (Figure 5D). The lower level of intracellular protein aggregation observed in the *ΔIDR* strain compared with the *Δdrhsp17* strain may reflect residual chaperone activity retained by the N-terminal α-crystallin domain of the *ΔIDR* variant. In addition, we performed in vitro protection assays using the total protein extract of the Δ*drhsp17* strain. Following acid stress, exogenous addition of wild-type DrHsp17 preserved a larger proportion of client proteins in the soluble fraction, as visualized by SDS-PAGE, whereas the addition of mutant variants exhibited a greater loss of protein bands from the soluble supernatant (Figure 5C), indicating that disruption of the C-terminal IDR and the accompanying loss of phase separation are associated with reduced chaperone protection under acid stress. This was further quantified by SYPRO Orange fluorescence, which is used as a reporter for unfolded proteins. The wild-type DrHsp17 markedly attenuated acid-induced protein unfolding in the cell extract, whereas all the mutants showed severely decreased protective capacities (Figure 5E).

Finally, we employed two model client proteins, β-galactosidase (GAL) and malate dehydrogenase (MDH), to evaluate the chaperone activity of DrHsp17 LLPS in vitro. Following pH 4.0 treatment, the wild-type DrHsp17 preserved higher relative enzymatic activities of both GAL (Figure 5F) and MDH (Figure 5G). In contrast, the phase-separation-deficient mutants showed a reduced ability to retain client enzyme function. Collectively, these in vivo and in vitro results show that disruption of the polyanionic C-terminal IDR is accompanied by loss of phase-separation capacity and reduced protection against acid-induced protein aggregation, unfolding, loss of solubility, and loss of enzymatic activity. These findings support an important contribution of the IDR-associated phase-separation behavior to the optimal chaperone function of DrHsp17 under acid stress, while not excluding LLPS-independent effects of the IDR mutations.

## 3. Discussion

### 3.1. pH-Dependent Transcriptional and Functional Responses of DrHsp17

The response of DrHsp17 to environmental pH is characterized by a notable separation between transcriptional induction and phenotypic requirement. Although *drhsp17* transcription was induced under several acidic conditions and showed a modest response under mild alkaline stress, the most evident functional contribution of DrHsp17 was observed under severe acid stress. This suggests that transcriptional responsiveness alone does not determine the physiological importance of DrHsp17 under a given pH condition. Instead, its protective function appears to involve an additional pH-sensitive regulatory layer at the protein level. Transient intracellular acidification has also been shown to function as a regulatory signal for the heat-shock response [34].

Bacterial acid-resistance systems classically act by limiting cytoplasmic acidification or reducing its immediate consequences, for example through energy-dependent proton extrusion, amino-acid-dependent buffering, envelope remodeling, and acid-activated molecular chaperones such as HdeA [4,5,6,7,8]. Previous studies have characterized heat-shock-responsive regulatory networks and sHsps in *D. radiodurans* [35,36]. In contrast, the acid-stress response of *D. radiodurans* remains poorly characterized despite its well-established capacity to tolerate severe macromolecular damage caused by ionizing radiation, oxidative stress, and desiccation [9,10]. The present results suggest that DrHsp17 may provide an additional layer of proteostasis control under severe acid stress. Rather than directly demonstrating a role in preventing intracellular acidification, our data indicate that DrHsp17 undergoes a rapid and reversible condensation response that is associated with reduced protein aggregation and improved preservation of protein function. We therefore propose that this phase-separation-associated proteostasis response may complement more conventional acid-homeostasis mechanisms and contribute to the robustness of *D. radiodurans* under acidic conditions. However, because direct cross-species comparisons of acid tolerance were not performed here, we do not attribute the environmental resilience of *D. radiodurans* solely to DrHsp17 or to this mechanism.

### 3.2. A Protonation-Based Model for the Polyanionic C-Terminal IDR-Driven Phase Separation of DrHsp17

A plausible molecular explanation for the pH-dependent phase behavior of DrHsp17 is based on the protonation of acidic residues within its C-terminal IDR. Under neutral conditions, the Asp/Glu-rich IDR is expected to carry a high net negative charge, which would disfavor intermolecular condensation through electrostatic repulsion. As the environmental pH decreases toward the apparent pKa range of acidic side chains, progressive protonation may reduce this repulsion and favor additional weak intermolecular interactions. Recent studies of D/E-rich condensates further suggest that protonation of acidic tracts can alter their interaction properties and local physicochemical environment [37], while pH-sensing regions have also been shown to regulate reversible protein assembly [38].

We therefore propose that changes in the protonation state of the polyanionic C-terminal IDR may contribute to acid-induced DrHsp17 condensation. It is important to note that the D/E-A mutant should not be regarded as a direct mimic of the protonated state of Asp/Glu residues. Although substitution with alanine removes the negative charges of these acidic residues, it also eliminates their carboxyl side chains and the associated polarity and hydrogen-bonding potential. Therefore, the inability of the D/E-A mutant to form condensates at physiological pH suggests that charge neutralization alone is insufficient to reproduce the molecular state of protonated acidic residues. One possibility is that, in addition to reducing electrostatic repulsion, protonated Asp/Glu residues contribute specific weak intermolecular interactions, including hydrogen bonding, that favor condensation. Substitution of Asp/Glu with the corresponding uncharged amides, Asn/Gln, would provide a more direct experimental test of this possibility and represents an important direction for future mechanistic studies. However, because neither intracellular pH nor the protonation state of the DrHsp17 IDR was directly measured in the present study, this mechanism should be regarded as a working model rather than a directly demonstrated molecular switch.

### 3.3. Acid-Induced Phase Separation as an Alternative Stress-Responsive Reorganization of DrHsp17

These findings also provide a possible answer to the mechanistic question raised in the Introduction regarding how an atypical, predominantly dimeric sHsp such as DrHsp17 can be reorganized under severe acid stress without relying on the classical oligomer-dissociation pathway. In canonical bacterial sHsps, exemplified by the *E. coli* IbpA/IbpB system, stress activation is commonly associated with remodeling of higher-order oligomeric assemblies into smaller, more substrate-accessible species [39,40]. By contrast, previous structural studies showed that DrHsp17 exists predominantly as an elongated dimer with its C-terminal regions exposed to the solvent [23]. Our present data suggest that acid-induced condensation provides an alternative mode of stress-responsive structural reorganization: rather than requiring dissociation from a large oligomeric reservoir, the exposed C-terminal IDRs of DrHsp17 can participate in intermolecular interactions that promote reversible condensate formation under acidic conditions. Such dynamic material properties are characteristic of stress-responsive biomolecular condensates and can be modulated by intermolecular interactions and protein quality-control factors [41,42].

This interpretation is further supported by our biochemical analyses. Native PAGE and size-exclusion chromatography showed no detectable acid-induced transition to a lower-molecular-weight soluble species for wild-type DrHsp17, D/E-A, or ΔIDR when condensates were disrupted with 1,6-hexanediol (Figure S10). In particular, the D/E-A mutant exhibited a soluble assembly profile similar to that of wild-type DrHsp17, arguing against a major mutation-induced disruption of the underlying sHsp assembly state. The residual chaperone activity retained by the D/E-A and ΔIDR variants further indicates that phase separation is not absolutely required for the basal chaperone function of DrHsp17. Rather, the data suggest that the C-terminal IDR and its associated phase-separation capacity enhance the efficiency of protein protection under acid stress. This interpretation is consistent with the presence of the conserved α-crystallin domain, which may retain a degree of substrate-binding or holdase activity even when the C-terminal IDR is altered. Therefore, LLPS is best viewed as a stress-responsive mechanism that augments, rather than constitutes the entirety of, DrHsp17 chaperone activity. Together, these observations support a model in which acid-induced LLPS represents an alternative stress-responsive reorganization of DrHsp17 that does not require the canonical oligomer-dissociation mechanism. The major differences between the canonical *E. coli* IbpA/IbpB system and DrHsp17 are summarized in Table 1. However, because the present experiments do not directly resolve all intermediate assembly states, this mechanism should be regarded as the interpretation most consistent with the available data rather than an exclusive activation pathway.

Nevertheless, although the variants displayed detectable intracellular fluorescence and showed no gross alteration in soluble assembly by Native PAGE and SEC, their in vivo protein abundance and turnover were not quantitatively measured. Therefore, subtle differences in expression level or stability among wild-type DrHsp17, ΔIDR, and D/E-A cannot be fully excluded as contributing factors to the observed phenotypes.

### 3.4. Physiological Significance and Potential Bioremediation Implications

The reversible and pH-dependent organization of DrHsp17 may provide *D. radiodurans* with a rapid proteostasis response that complements transcriptional and conventional acid-homeostasis mechanisms. Such a reversible physical reorganization may be particularly advantageous during transient or fluctuating acidic stress, because it can potentially respond on a timescale faster than de novo protein synthesis. This feature may be relevant to the persistence of *D. radiodurans* in acidic, metal- and radionuclide-contaminated environments and may also inform future engineering strategies aimed at improving the robustness of this organism for bioremediation applications [43,44,45].

Nevertheless, the present study does not establish that DrHsp17-mediated condensation alone accounts for the acid resistance of *D. radiodurans*. Rather, it should be considered one component of a broader stress-protection network operating in this extremophilic bacterium.

## 4. Materials and Methods

### 4.1. Bacterial Strains and Culture Conditions

All bacterial strains and plasmids used in this study are listed in Table S1. *Escherichia coli* strains, including the cloning host DH5α and the expression host BL21 (DE3), were routinely cultivated at 37 °C in Luria–Bertani (LB) broth (1% tryptone, 0.5% yeast extract and 1% NaCl). For solid media preparation, 1.5% agar was added to the LB broth.

*D. radiodurans* strains, including the wild-type R1 and its derivatives, were propagated at 30 °C in tryptone-glucose-yeast extract (TGY) broth (0.5% tryptone, 0.1% glucose and 0.3% yeast extract). Solid TGY plates were prepared by the addition of 1.5% agar.

When required, kanamycin was used at 30 μg/mL for selection of the *D. radiodurans Δdrhsp17* mutant and at 50 μg/mL for maintenance of pET28aT5-derived plasmids in *E. coli*, whereas chloramphenicol was used at 4 μg/mL for selection and maintenance of pRADP1-derived plasmids in *D. radiodurans*.

### 4.2. Generation of Mutant and Recombinant Strains

The primers used in this study are listed in Table S2. The *drhsp17* deletion mutant (Δ*drhsp17*) was generated by an optimized tripartite ligation strategy based on homologous recombination. Briefly, the upstream and downstream homologous flanking regions of the *drhsp17* gene were PCR-amplified from the genomic DNA of the *D. radiodurans* wild-type strain using the specific primer pairs *Δdrhsp17*-p1/p2 and *Δdrhsp17*-p3/p4, respectively. The upstream and downstream fragments were digested with appropriate restriction enzymes (*Bam*HI and *Hind*III) (Takara Bio, Kyoto, Japan) and subsequently ligated to a kanamycin resistance cassette using T4 DNA ligase.

The tripartite ligation product was directly transformed into competent *D. radiodurans* wild-type cells. Mutant transformants were selected on TGY agar plates supplemented with 30 μg/mL kanamycin. Targeted gene disruption was confirmed by genomic PCR using the primers *Δdrhsp17*-p1/p4 and *Δdrhsp17*-p5/p6. Confirmed deletion mutants were stored at −80 °C for subsequent assays.

For the construction of overexpression and complementation strains, the sequence of full-length wild-type *drhsp17*, the IDR-truncated mutant (*ΔIDR*), and the charge-altered mutants (*D/E-A*) were amplified using *P1-drhsp17-1* and their corresponding specific reverse primer (*P1-drhsp17-2*, *P1-ΔIDR-2* and *P1-D/E-A-2*). To visualize intracellular DrHsp17, these target fragments were cloned into the *E. coli*-*D. radiodurans* shuttle vector pRADP1 fused with an N-terminal mGFP using the *P1-mGFP-drhsp17-1* as a forward primer instead of *P1-drhsp17-1*. The resulting recombinant plasmids were transformed into the *Δdrhsp17* mutant or wild-type strains to obtain the required functional complementation or overexpression strains. The pRAD-derived expression system used in this study was previously developed and characterized in our laboratory and has been validated for heterologous protein expression in *D. radiodurans* at both transcriptional and translational levels [46]. The resulting recombinant plasmids carrying wild-type *drhsp17*, *ΔIDR*, or *D/E-A* were constructed using the same vector backbone and promoter and transformed into the corresponding *D. radiodurans* strains.

### 4.3. Survival and Phenotypic Analyses

The *D. radiodurans* wild-type and all mutant strains were cultivated in TGY liquid medium at 30 °C with shaking at 220 rpm until the OD_600_ reached 1.0. Subsequently, 1 mL of each bacterial culture was harvested by centrifugation at 3000× *g* for 5 min. The supernatant was discarded, and the cell pellets were washed once with 1× phosphate-buffered saline (PBS).

For the pH stress treatments, the washed cells were resuspended in pH stress buffer (40 mM Na_2_HPO_4_, 20 mM citric acid, 137 mM NaCl, 2.7 mM KCl) that had been adjusted to specific pH values (pH 4.0, 5.0, 6.0, 7.0, and 8.0). The cell suspensions were incubated under these conditions for designated time intervals (0, 1, 2 h). For survival phenotypic analysis under oxidative, UV radiation, or heat stresses, the cells were exposed to oxidative stress with H_2_O_2_ (0, 30, and 50 mM), UV radiation (0, 300, and 450 J/m^2^), or 48 °C heat treatment (0, 1, and 2 h), respectively.

Following the treatments, the bacterial suspensions were serially diluted 10-fold using sterile 1× PBS to obtain a dilution series (10^−1^ to 10^−6^). For survival fraction assays, 50 μL of the diluted bacterial solution was applied to TGY agar plates for counting. For the phenotypic assays, 10 μL of each corresponding dilution was spotted onto TGY agar plates. The plates were incubated upside down at 30 °C for 2 to 3 days to allow for visible colony formation. No antibiotics were supplemented during the cultivation and the subsequent stress assays to exclude any confounding impacts on cell viability. All viability and phenotypic assays were performed in three independent biological replicates.

### 4.4. Real-Time Quantitative PCR

Gene expression under stress was analyzed by RT-qPCR. The *D. radiodurans* wild-type strain was cultured in TGY medium to an OD_600_ of 1.0 and subsequently subjected to stress. After the designated treatment, 10 mL aliquots of bacterial culture were harvested by centrifugation at 8000× *g* for 2 min. The supernatant was discarded, and the cell pellets were resuspended in 200 μL of 20 mg/mL lysozyme solution (in DEPC-treated water) and incubated at 37 °C for 30 min for cell lysis. Total RNA was extracted using the TransZol Up Plus RNA Kit (TransGen, Beijing, China). For cDNA synthesis, 1 μg of total RNA was reverse-transcribed using the TransScript All-in-One First-Strand cDNA Synthesis SuperMix for qPCR (One-Step gDNA Removal) Kit (TransGen, Beijing, China). RT-qPCR amplification was performed on an Mx3005P real-time PCR detection system (Stratagene, San Diego, CA, USA) using the TB Green Fast qPCR Mix (Takara Bio, Kyoto, Japan). Primer sequences for target and reference genes are listed in Table S2. Relative transcript levels were calculated using the 2^−ΔΔCt^ method, with the *dr_1343* (encoding glyceraldehyde-3-phosphate dehydrogenase, GAPDH) serving as the endogenous control to normalize expression levels. All biological experiments were performed in independent triplicates. Following amplification, melting-curve analysis was performed to assess product specificity.

### 4.5. Protein Expression and Purification

The sequences encoding wild-type DrHsp17, its variants (ΔIDR and D/E-A), and the mGFP-DrHsp17 fusion protein were amplified by PCR using the specific primers listed in Table S2. The amplified target genes were subsequently cloned into the pET28aT5 expression vector, appending an N-terminal 6×His-tag sequence to the encoded proteins.

The recombinant plasmids were transformed into *E. coli* BL21 (DE3) competent cells. Transformants were grown aerobically in LB liquid medium supplemented with 50 μg/mL kanamycin at 37 °C until the OD_600_ reached 0.6–0.8. Protein expression was induced by the addition of p-Isopropyl benzoic acid (Cuminic acid) to a final concentration of 50 μg/mL, followed by further incubation at 18 °C for 16 h.

Following induction, the cells were harvested by centrifugation and resuspended in lysis buffer (20 mM Tris-HCl, pH 8.0; 500 mM NaCl; 5% (*w*/*v*) glycerol), and lysed by sonication. Insoluble debris was removed by centrifugation at 15,000× *g* for 30 min at 4 °C.

The clarified supernatants were loaded onto a Ni-NTA affinity column (GE Healthcare, Chicago, IL, USA) pre-equilibrated with lysis buffer. The column was washed with buffer containing 25 mM imidazole to eliminate non-specifically bound impurities, and the target His-tagged proteins were subsequently eluted using a high-imidazole (500 mM) buffer. For final polishing and buffer exchange, the eluted fractions were concentrated and subjected to size-exclusion chromatography using a Superdex 200 column (GE Healthcare, Chicago, IL, USA) equilibrated with a final storage buffer (20 mM Tris-HCl, pH 7.5; 150 mM KCl). Protein purity and molecular weights were verified by SDS-PAGE. Purified protein fractions were aliquoted and stored at −80 °C for subsequent assays.

### 4.6. Subcellular Localization and Fluorescence Imaging

*D. radiodurans* cells expressing mGFP-fused proteins were harvested at the exponential growth phase (OD_600_ = 1.0) and subjected to pH stress buffers at pH 4.0 and 7.0. Subsequently, 1 mL of the bacterial culture was centrifuged at 3000× *g* for 5 min to collect the bacterial cells. After washing with 1× PBS, 40 μL of 1× PBS was added to resuspend the cells. A 5 μL aliquot of the treated cell suspension was then deposited onto a clean glass slide covered with a coverslip and observed using an inverted fluorescence microscope, Eclipse Ti2 (Nikon, Tokyo, Japan). For the in vivo dissolution assay, 1,6-hexanediol was added to the cell suspension to a final concentration of 12.5% prior to imaging. For the dead cell staining assay, cell suspension was further incubated with EthD-III from Live-Dead Bacterial Staining Kit (APExBio, Huston, TX, USA) according to the product protocol before imaging. For quantitative analysis of intracellular puncta, fluorescence images were collected from independent biological experiments under identical imaging settings. The number of discrete fluorescent puncta in each cell of the whole image was quantified using ImageJ 1.54m.

### 4.7. In Vitro LLPS Analysis

Purified proteins were mixed with LLPS buffer (20 mM Na_2_HPO_4_, 20 mM citric acid, 150 mM KCl) at pH 4.0 to 8.0 in a total volume of 20 μL. The protein mixtures were then transferred onto a glass coverslip. The formation of liquid droplets was observed using Elipse Ti2. For the in vitro dissolution assay, 1,6-hexanediol was introduced into the system at final concentrations of 4% or 8%.

### 4.8. Fluorescence Recovery After Photobleaching (FRAP)

The FRAP experiments were conducted using a Leica TCS SP8 confocal laser scanning microscope (Leica Microsystems, Wetzlar, Germany). Briefly, the mGFP-DrHsp17 fusion protein was induced to phase separate (30 μM protein in LLPS buffer, pH 4.0), and the droplets were visualized using a 100× oil-immersion objective. The FRAP procedure was executed using the dedicated FRAP module within the LAS X 3.3.0.16799 software. Initially, 39 pre-bleach frames were acquired at a low laser intensity (0.5%). Subsequently, a circular region of interest (ROI) was defined at the center of a target droplet and photobleached using 50% laser power (488 nm) for 13 iterations. The fluorescence recovery within the ROI was continuously monitored at 0.5% laser intensity for 470 s. The fluorescence intensity of the bleached ROI was extracted and normalized against the fluorescence of an unbleached reference droplet area using the LAS X analysis software.

### 4.9. Turbidity Assays

The turbidity of the protein solutions was assessed by measuring the optical absorbance at 405 nm using an Eppendorf BioSpectrometer (Eppendorf, Hamburg, Germany). Purified proteins were mixed thoroughly with LLPS buffer at pH 4.0 to 8.0 in a total volume of 50 μL for each measurement. For each condition, the corresponding buffer devoid of protein was used as a blank. Turbidity measurements were performed in three independent experiments for each condition, and the data are presented as mean ± SD.

### 4.10. Analysis of Chaperone Activity

The preparation of protein extracts of *Δdrhsp17* was performed following the method described previously [47]. Then, the protein extract (5.0 mg/mL) was incubated with the purified wild-type DrHsp17 or its mutant variants (0.6 mg/mL) in a total volume of 60 μL under acid stress (pH 4.0) or neutral conditions (pH 7.0). After the incubation, the supernatants were collected and analyzed using SDS-PAGE to visualize the retained soluble proteins.

For the SYPRO Orange fluorescence assay, *Δdrhsp17* protein extract (1.0 mg/mL) with wild-type DrHsp17 or its mutant variants (0.6 mg/mL) was treated with acid stress and then incubated with 2× SYPRO Orange dye. Fluorescence was then read at 470 nm excitation and 570 nm emission using a SpectraMax iD5 plate reader (Molecular Devices, Sunnyvale, CA, USA). The relative fluorescence increase was calculated by comparing the acid-stressed samples to their respective pH 7.0 controls.

For the ThT fluorescent assays, the *D. radiodurans* wild-type, *Δdrhsp17* mutant, and complemented strains were cultured to an OD_600_ of 1.0 and subsequently exposed to pH stress buffer at pH 4.0 or pH 7.0. The cells were then collected by centrifugation, washed once with 1× PBS, and resuspended in 1× PBS containing 50 μM ThT, followed by incubation for 45 min. Fluorescence was read at 440 nm excitation and 482 nm emission.

The GAL and MDH protection assays were carried out at 30 °C with a total volume of 100 μL of reaction mix. Substrate GAL (0.2 mg/mL) was incubated with wild-type DrHsp17 or its mutant variants (0.3 mg/mL) for 1 h in reaction buffer (5× Na_2_HPO_4_-citric acid buffer; 150 mM KCl; 0.1 mM FDG). Then, the fluorescence of Ex/Em = 490/520 nm was measured. Substrate MDH (1.0 mg/mL) was incubated with wild-type DrHsp17 or its mutant variants (0.3 mg/mL) for 30 min in reaction buffer (5× Na_2_HPO_4_-citric acid buffer; 150 mM KCl; 5 mM L-malate; 0.5 mM NAD^+^). The optical absorbance at 340 nm was measured. Before the measurement, 5 μL of 1 M Tris-HCl pH 8.0 was added to disrupt the phase-separated droplets. The relative retained activities of GAL and MDH were calculated by normalizing the activity measured after pH 4.0 treatment to that of the corresponding sample under pH 7.0 conditions, according to the following formula: relative retained activity (%) = (activity at pH 4.0/activity at pH 7.0) × 100.

### 4.11. Statistical Analysis

Statistical analyses were performed using GraphPad Prism 9.5.1. Data are presented as the mean ± standard deviation (SD) from the indicated number of independent biological experiments. For comparisons involving three or more groups, ordinary one-way ANOVA followed by Dunnett’s multiple-comparisons test was used to compare each indicated group with the designated control group. For experiments involving multiple time points or treatment conditions, one-way ANOVA was performed separately for each indicated time point or condition, as specified in the corresponding figure legends. Statistical significance was defined as *p* < 0.05; *, *p* < 0.05; **, *p* < 0.01; ***, *p* < 0.001; ****, *p* < 0.0001; ns, not significant.

## 5. Conclusions

In summary, this study identifies DrHsp17 as an acid-responsive small heat shock protein that contributes to the survival and proteostasis of *D. radiodurans* under severe acidic conditions. DrHsp17 undergoes a rapid and reversible pH-dependent condensation response in vivo and in vitro, and this behavior requires its polyanionic C-terminal IDR under the tested conditions. Disruption of this region abolishes condensate formation and is accompanied by reduced protection against acid-induced protein aggregation, unfolding, loss of solubility, and loss of enzymatic activity. These findings support an important contribution of the C-terminal IDR and its associated phase-separation behavior to the optimal protective function of DrHsp17.

Several mechanistic questions remain unresolved. Direct measurement of the protonation state of DrHsp17 C-terminal IDR, for example by NMR-based pH titration, would help test the proposed protonation-dependent model of condensation. Real-time monitoring of intracellular pH in *D. radiodurans* would further clarify the physiological pH range that triggers DrHsp17 condensation in vivo. Future studies examining client partitioning and the physicochemical properties of DrHsp17 condensates will also be important for defining how phase separation contributes to protein protection under acid stress. Overall, our findings reveal a distinct mode of stress-responsive reorganization for an atypical bacterial sHsp and expand the current understanding of proteostasis regulation in *D. radiodurans* under acid stress.

## Figures and Tables

**Table 1:** Comparison of stress-responsive organization of canonical bacterial sHsps and DrHsp17.

Feature	*E. coli* IbpA/IbpB	*D. radiodurans* DrHsp17
Basal assembly state	higher-order oligomeric assemblies	predominantly elongated dimer
Stress-responsive reorganization	oligomer remodeling/dissociation into smaller active species	acid-induced reversible condensation/LLPS
Major structural determinant	oligomeric interfaces and α-crystallin-domain-associated assembly	polyanionic C-terminal IDR
Proposed activation logic	increased substrate accessibility after oligomer remodeling	pH-responsive intermolecular interactions promote condensation

## Data Availability

All data supporting the findings of this study have been provided in the manuscript and its Supplementary Materials. Correspondence and requests for materials should be addressed to the authors.

## Supplementary Materials

The following supporting information can be downloaded at: https://www.mdpi.com/article/10.3390/ijms27219801/s1.
