# Supplementary material for: Polyanionic C-Terminal IDR-Dependent Phase Separation of DrHsp17 Contributes to Acid Stress Protection in Deinococcus radiodurans
Source: Int J Mol Sci. 2026 Oct 31;27(21):9801. doi: 10.3390/ijms27219801 (PMC13762983; doi:10.3390/ijms27219801)
Supplement: Supplementary file 1 [file ijms-27-09801-s001.zip › ijms-4565927-supplementary.pdf]

Supplementary information

Polyanionic C-Terminal IDR-Dependent Phase Separation of DrHsp17 Contributes to Acid Stress Protection in *Deinococcus radiodurans*

Ning Yu[1], Shang Dai[2], Binqiang Wang[1], Chunhui Cai[1], Liangyan Wang[1], Yuejin Hua[1], Bing Tian[1,3,*]

1. Institute of Biophysics, College of Life Sciences, Zhejiang University, Hangzhou 310058, China
2. College of Life Sciences, Nanjing Agricultural University, Nanjing 21095, China
3. Zhejiang University-Lishui Joint Innovation Center for Life and Health & Lishui Lvgu Institute for Life and Health, Lishui 323010, China

* Corresponding author. Email: tianbing@zju.edu.cn (B.T.)

Supplementary Figures

Supplementary Figure S1. Construction and PCR verification of the *drhsp17* deletion mutant

Supplementary Figure S2. Complementation of *drhsp17* restores the acid stress resistance of the *Δdrhsp17* mutant

Supplementary Figure S3. Survival phenotypic analysis and transcriptional response of *drhsp17* under mild acid and alkaline conditions

Supplementary Figure S4. Representative melting-curve analysis of RT-qPCR products for the reference gene *dr_1343* and target gene *drhsp17*

Supplementary Figure S5. Survival phenotypic analysis and transcriptional response of *drhsp17* under oxidative, UV radiation, and heat stresses

Supplementary Figure S6. Sequence alignment of DrHsp17 with homologous proteins

Supplementary Figure S7. Viability assessment of *D. radiodurans* cells exhibiting mGFP-DrHsp17 condensates under acid stress

Supplementary Figure S8. SDS-PAGE analysis of the purified recombinant DrHsp17 and its variant proteins

Supplementary Figure S9. *In vitro* analysis of DrHsp17 and the mGFP-DrHsp17 fusion protein LLPS

Supplementary Figure S10. Biochemical characterization of the oligomeric states of DrHsp17 and its variants under neutral and acidic conditions

Supplementary Tables

Supplementary Table S1. Strains and plasmids used in this study

Supplementary Table S2. Primers used in this study

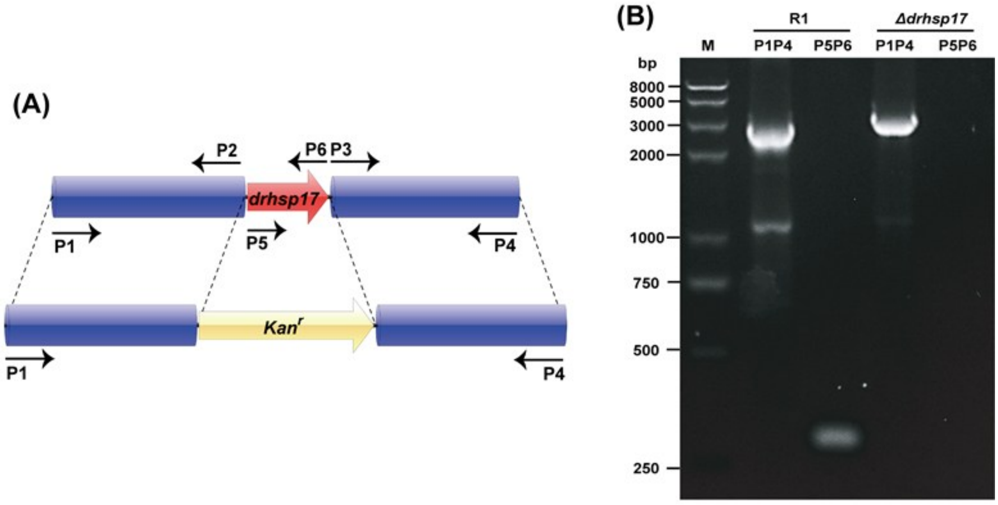

Figure S1. Construction and PCR verification of the *drhsp17* deletion mutant. **(A)** Schematic representation of the targeted gene deletion strategy via homologous recombination. The coding sequence of the *drhsp17* was replaced by a kanamycin resistance cassette (*Kan*^{r}). The orientations of the primers (P1 to P6) used for mutant construction and subsequent PCR verification are indicated by black arrows. **(B)** PCR confirmation of the *Δdrhsp17* mutant using agarose gel electrophoresis. Lane M, the DNA molecular weight marker.

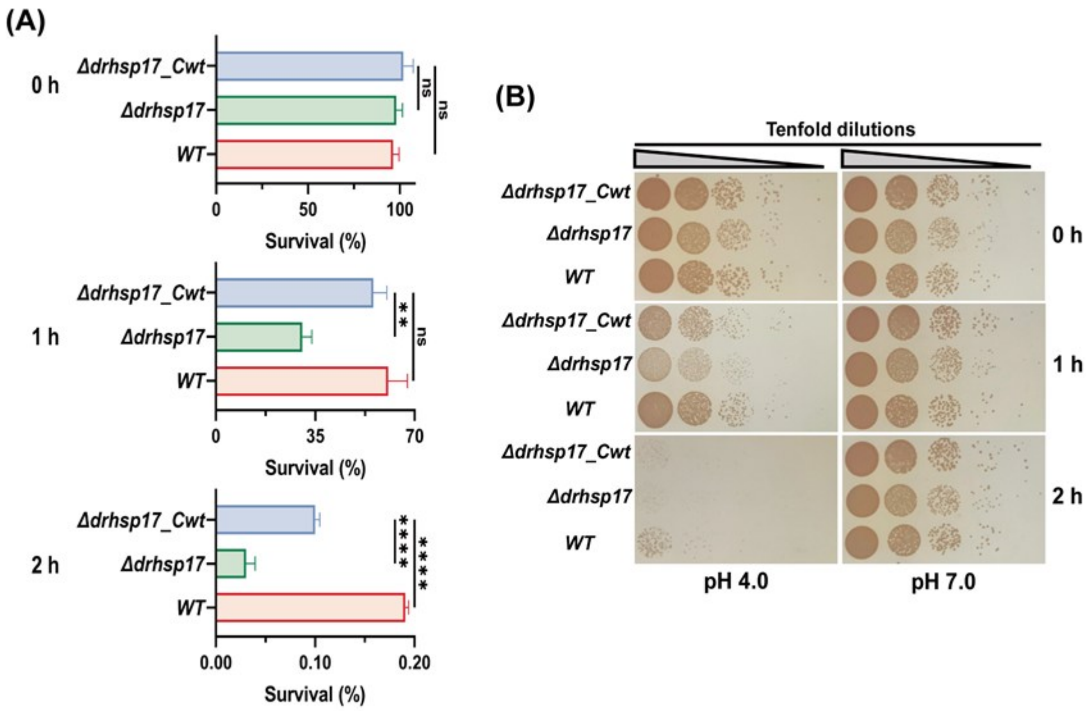

Figure S2. Complementation of *drhsp17* restores the acid stress resistance of the *Δdrhsp17* mutant. **(A)** Survival fractions (%) of the wild-type (WT), *drhsp17* deletion (*Δdrhsp17*), and complementation (*Δdrhsp17_Cwt*) strains after exposure to pH 4.0 acid stress for 0, 1, and 2 h. **(B)** Survival phenotypic assays of the WT, *Δdrhsp17*, and *Δdrhsp17_Cwt* strains under acid stress (pH 4.0). Cells were treated with pH stress buffer (40 mM Na_2HPO_4, 20 mM citric acid, 137 mM NaCl, 2.7 mM KCl) at pH 4.0 or pH 7.0 control for 0, 1, and 2 h, respectively. Tenfold serial dilutions of the cultures following acid stress treatments were spotted onto agar plates. Data in (A) are presented as mean ± SD of three independent biological replicates. Statistical significance was determined by one-way ANOVA followed by Dunnett's multiple-comparisons test. **, $p < 0.01$; ****, $p < 0.0001$; ns, not significant.

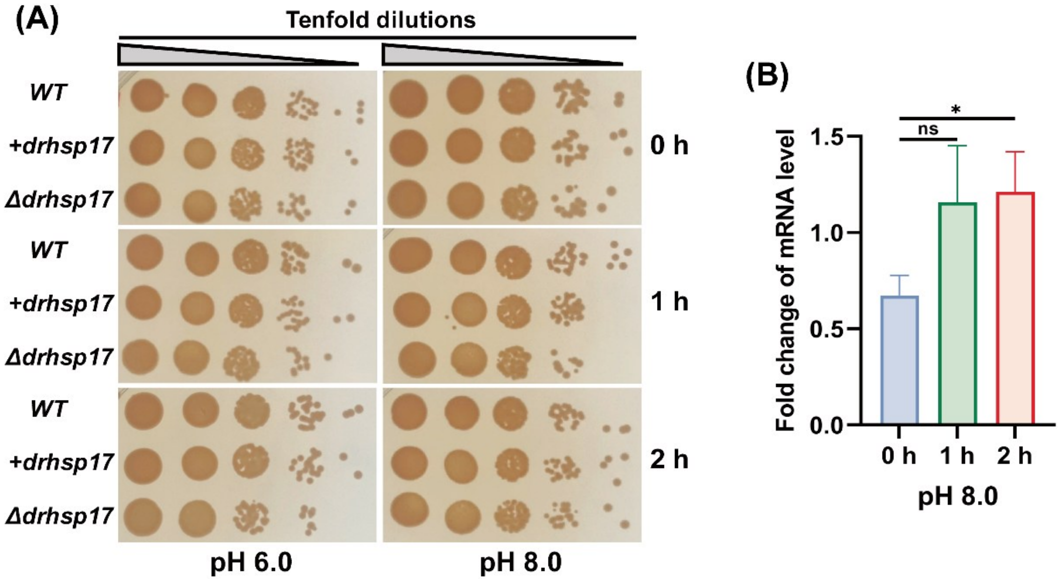

Figure S3. Survival phenotypic analysis and transcriptional response of *drhsp17* under mild acid and alkaline conditions. **(A)** Survival phenotypic assays of the *D. radiodurans* wild-type, *drhsp17* overexpression (+*drhsp17*), and deletion (*Δdrhsp17*) strains. Cultures were exposed to mildly acidic (pH 6.0) or mildly alkaline (pH 8.0) buffers for 0, 1, and 2 h, respectively. **(B)** Relative mRNA expression levels of *drhsp17* in the wild-type strain under mild alkaline stress (pH 8.0). Transcript levels were quantified by RT-qPCR after 0, 1, or 2 h of treatment. Data are presented as the mean ± standard deviation (SD) of three independent biological replicates. Statistical significance was analyzed using one-way ANOVA followed by Dunnett's multiple-comparisons test. ns, not significant; *, $p < 0.05$.

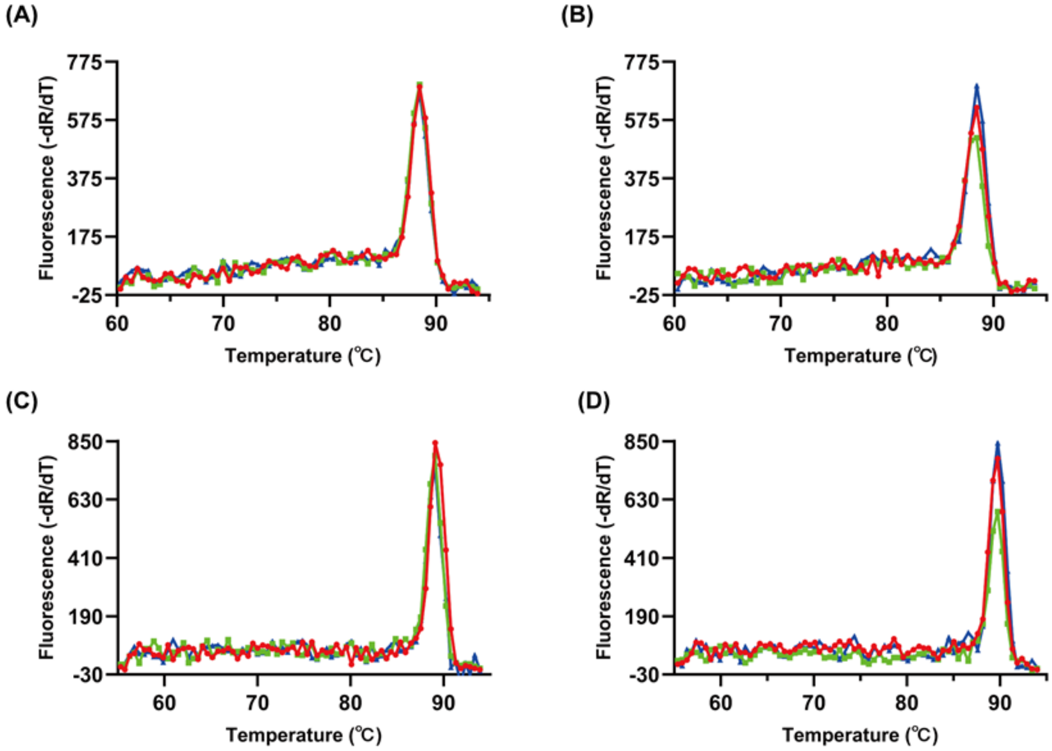

Figure S4. Representative melting-curve analysis of RT-qPCR products for the reference gene *dr_1343* and target gene *drhsp17*. **(A, B)** Melting curves of the RT-qPCR products amplified from the reference gene *dr_1343* under pH 7.0 control conditions (A) and pH 4.0 acid-stress conditions (B). **(C, D)** Melting curves of the RT-qPCR products amplified from the target gene *drhsp17* under pH 7.0 control conditions (C) and pH 4.0 acid-stress conditions (D). Each assay produced a single predominant melting peak without detectable secondary peaks, supporting the specificity of the corresponding primer pairs and the presence of a single major amplification product. Curves from three technical replicate reactions are shown for each condition.

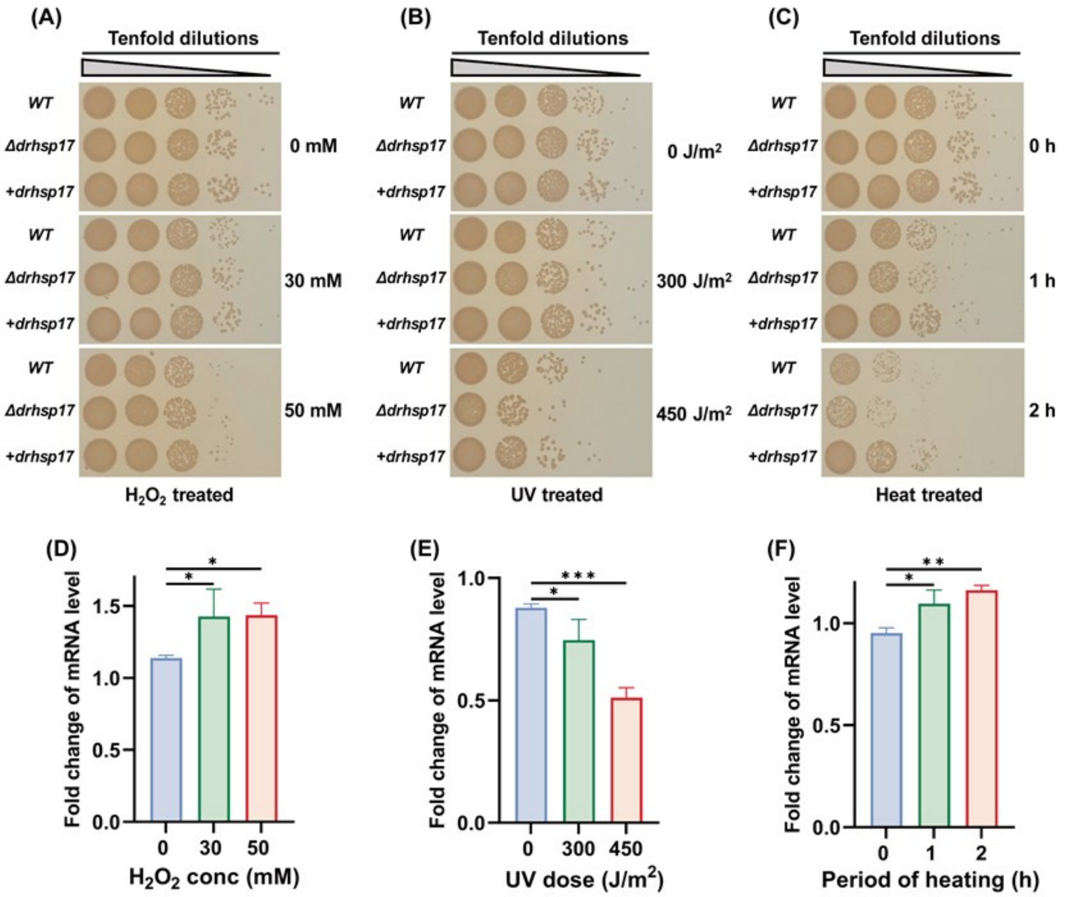

Figure S5. Survival phenotypic analysis and transcriptional response of *drhsp17* under oxidative, UV radiation, and heat stresses. **(A–C)** Survival phenotypic assays of the *D. radiodurans* wild-type (WT), *drhsp17* deletion (*Δdrhsp17*), and *drhsp17* overexpression (+*drhsp17*) strains. Cultures were exposed to oxidative stress with H_2O_2 (0, 30, and 50 mM) (A), UV radiation (0, 300, and 450 J/m^2) (B), and 48 ℃ heat treatment (0, 1, and 2 h) (C). Tenfold serial dilutions of the treated cultures were spotted onto agar plates. **(D–F)** Relative mRNA expression levels of *drhsp17* in the wild-type strain under the conditions corresponding to (A–C). Transcript levels were quantified by RT-qPCR. Data in (D–F) are presented as the mean ± standard deviation (SD) of three independent biological replicates. Statistical significance was analyzed using one-way ANOVA followed by Dunnett's multiple-comparisons test. *, $p < 0.05$; **, $p < 0.01$; ***, $p < 0.001$.

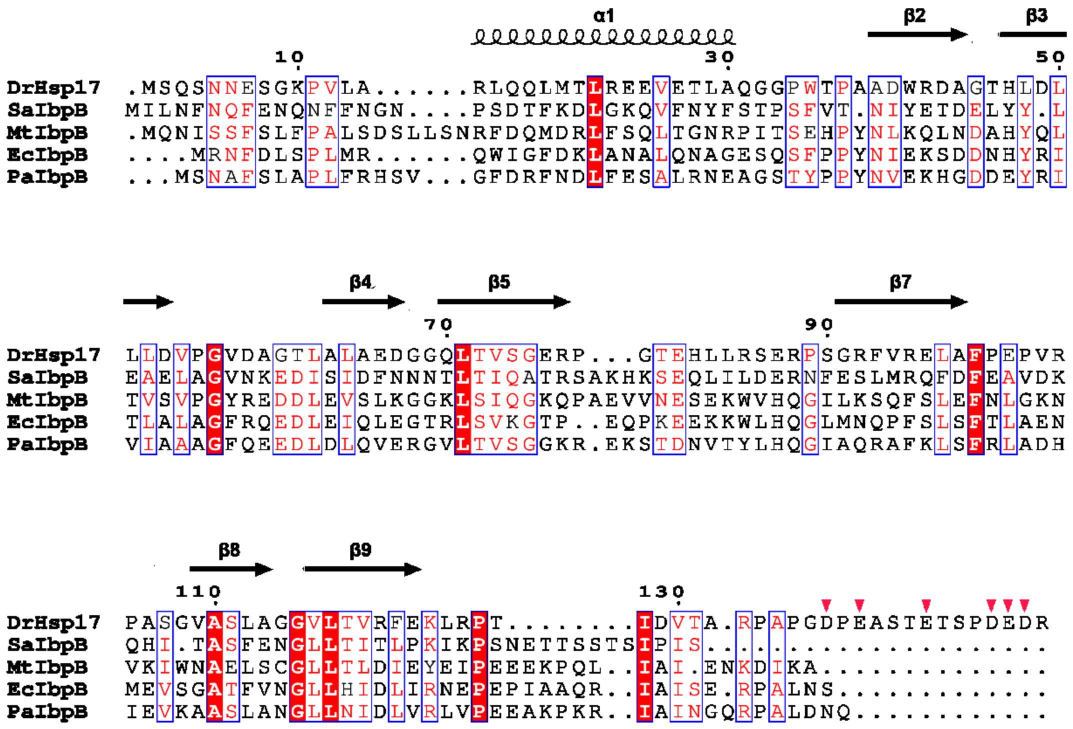

Figure S6. Sequence alignment of DrHsp17 with homologous proteins. The amino acid sequence of *D. radiodurans* DrHsp17 was aligned with its homologs from *S. aureus* (SaIbpB), *M. tuberculosis* (MtIbpB), *E. coli* (EcIbpB), and *P. aeruginosa* (PaIbpB). Strictly conserved residues are highlighted with a red background, and similar residues are shown within blue frames. Secondary structural elements (α-helix and β-strands) corresponding to DrHsp17 are indicated above the alignment sequences. The red arrows indicate the native negatively charged amino acids located within the C-terminal IDR of DrHsp17.

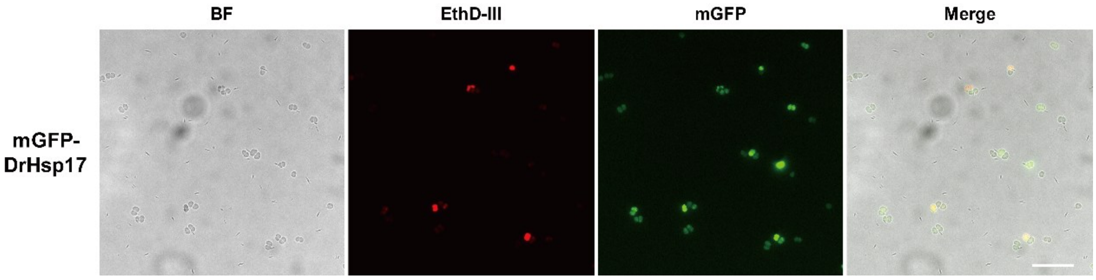

Figure S7. Viability assessment of *D. radiodurans* cells exhibiting mGFP-DrHsp17 condensates under acid stress. Fluorescence microscopy images of cells expressing mGFP-DrHsp17 following exposure to pH 4.0 buffer for 1 h. Cells were stained with Ethidium Homodimer III (EthD-III, red) to selectively identify dead cells. The distinct mGFP-DrHsp17 fluorescent puncta (green) are observed in viable cells (EthD-III negative). BF, bright field. Scale bar, 5 μm.

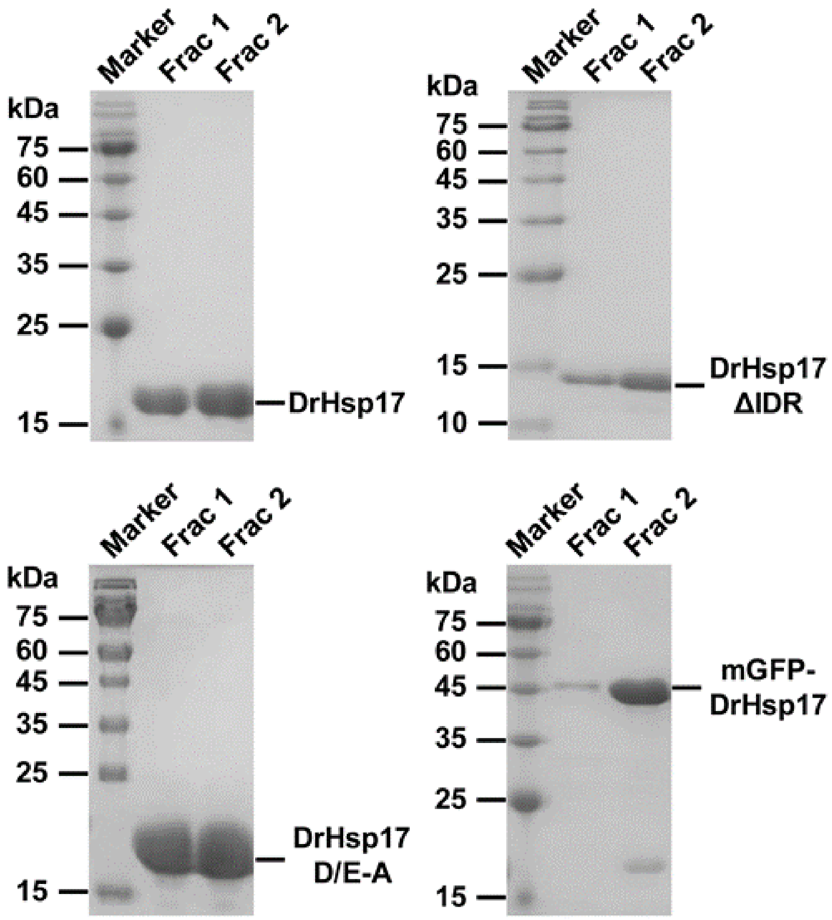

Figure S8. SDS-PAGE analysis of the purified recombinant DrHsp17 and its variant proteins**.** DrHsp17, the wild-type DrHsp17; DrHsp17 ΔIDR, truncated mutant lacking the intrinsically disordered region; DrHsp17 D/E-A, charge-altered site mutants; mGFP-DrHsp17, the mGFP-fused protein.

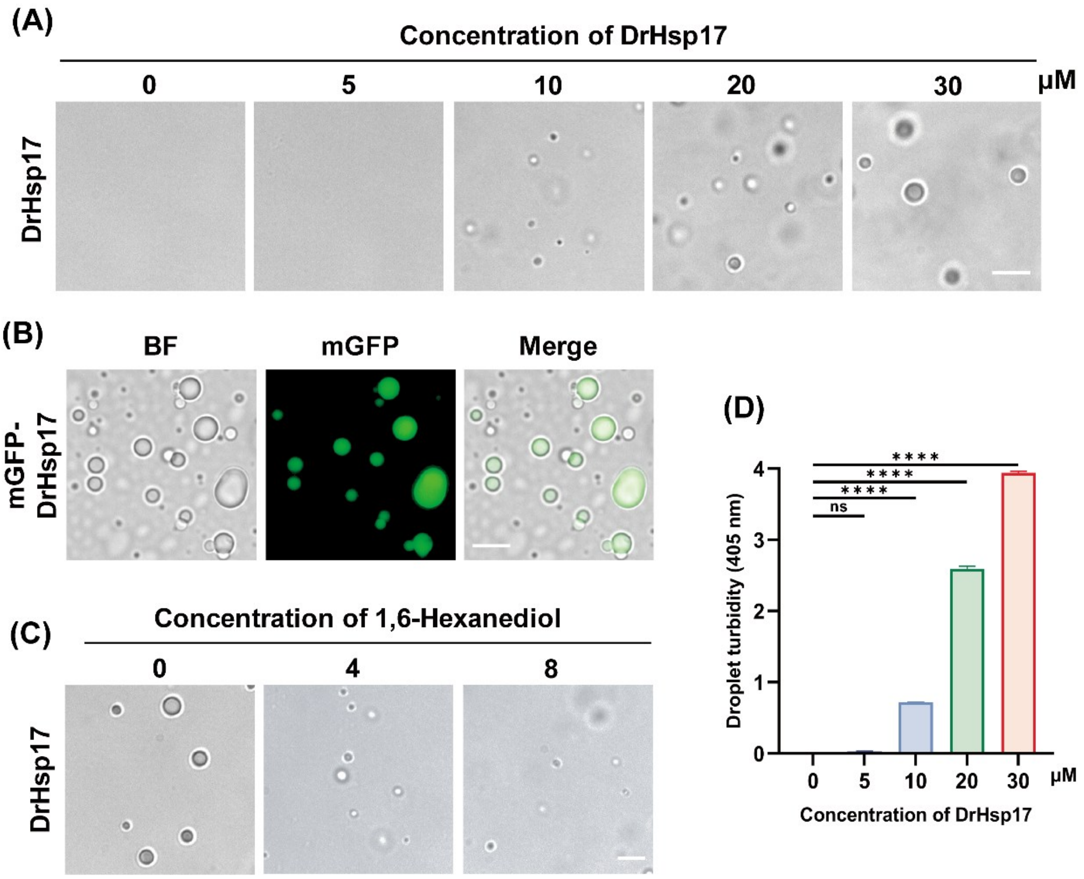

Figure S9. *In vitro* analysis of DrHsp17 and the mGFP-DrHsp17 fusion protein LLPS. All *in vitro* assays in this figure were performed in 2× Na_2HPO_4-citric acid buffer (20 mM Na_2HPO_4, 10 mM citric acid) at pH 4.0. **(A)** Representative microscopy images demonstrating the phase separation of DrHsp17 at varying protein concentrations (0, 5, 10, 20, and 30 μM) in pH 4.0. Scale bar, 5 μm. **(B)** Fluorescence microscopy images of the purified mGFP-DrHsp17 fusion protein (20 μM) under pH 4.0. BF, bright field. Scale bar, 10 μm. **(C)** Dissolution of DrHsp17 droplets (20 μM) *in vitro* following the addition of increasing concentrations of 1,6-hexanediol (0%, 4%, or 8%). Scale bar, 5 μm. **(D)** Turbidity assay of the DrHsp17 solutions as measured absorbance at 405 nm at the indicated concentrations corresponding to (A). Data are presented as the mean ± standard deviation (SD) of three independent replicates. Statistical significance was determined using one-way ANOVA followed by Dunnett's multiple-comparisons test. ****, $p < 0.0001$; ns, not significant.

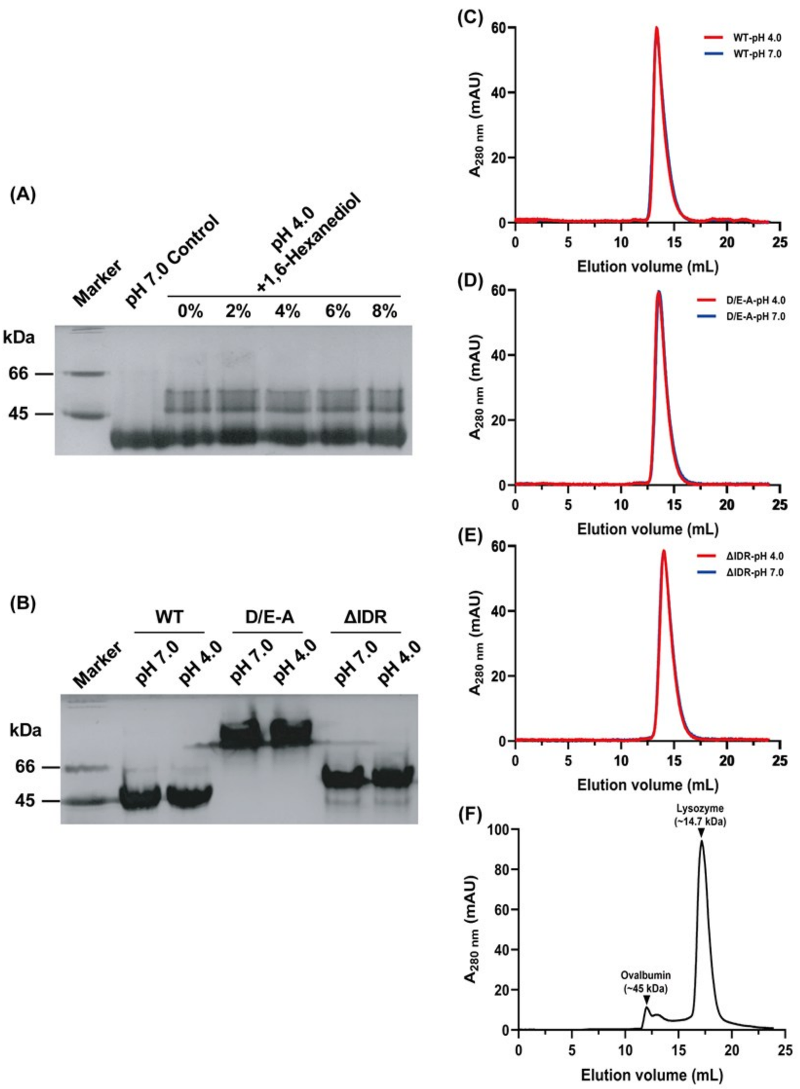

Figure S10. Biochemical characterization of the oligomeric states of DrHsp17 and its variants under neutral and acidic conditions. **(A)** Native PAGE analysis of purified wild-type DrHsp17 under neutral conditions (pH 7.0) or acidic conditions (pH 4.0) supplemented with increasing concentrations of 1,6-hexanediol (0%, 2%, 4%, 6%, and 8%). **(B)** Native PAGE analysis of purified wild-type DrHsp17, D/E-A, and ΔIDR proteins. Proteins (0.6 mg/mL) were separately incubated in pH 7.0 buffer or in pH 4.0 buffer supplemented with 6% 1,6-hexanediol before electrophoresis. **(C–E)** Size-exclusion chromatography (SEC) profiles of wild-type DrHsp17 (C), D/E-A (D), and ΔIDR (E) under the same conditions as in (B), using a Superdex 200 Increase 10/300 GL column and elution buffer (20 mM Na_2HPO_4, 20 mM citric acid, 150 mM KCl, 6% 1,6-hexanediol). Protein elution was monitored by absorbance at 280 nm. Red and blue curves represent the pH 4.0 and pH 7.0 conditions, respectively. **(F)** SEC profile of the molecular weight standards ovalbumin (~45 kDa, 0.1 mg/mL) and lysozyme (~14.7 kDa, 1 mg/mL), analyzed using the same column and elution buffer of pH 7.0 in (C–E). The theoretical molecular weights of the purified recombinant monomeric DrHsp17, D/E-A, and ΔIDR are 18.494, 18.188, and 17.079 kDa, respectively. Comparison of the elution volumes of these proteins with those of the molecular weight standards suggests that their apparent hydrodynamic sizes are more consistent with larger assemblies than with monomeric species of their theoretical molecular weights.

Table S1. Strains and plasmids used in this study

Strains	Relevant characteristics	Reference or source
D. radiodurans		
DraR1 wt	Wild-type strain ATCC13939	Laboratory stock
Δdrhsp17	R1 but *drhsp17::kan*	This study
Escherichia coli		
DH5α	Cloning strain	TOLOBIO
BL21 (DE3)	Expression strain	TOLOBIO
plasmids		
pET28aT5	T5 promoter, T7 terminator, Kan^r,	Laboratory stock
pET28aT5-mGFP	pET28aT5 with N-terminal mGFP	Laboratory stock
pRADP1	*E. coli-D. radiodurans* shuttle vector	Laboratory stock
pRADP1-mGFP	pRADP1 with N-terminal mGFP fusion	Laboratory stock

Table S2. Primers used in this study

Primers	Sequence (5′-3′)
Expression of proteins	
DrHsp17-1	GAAAACCTGTATTTTCAGGGCCATATGTCGCAGAGCAACAA
DrHsp17-2	CGGAGCTCGAATTCGGATCCTCAGCGGTCTTCGTCAGGAC
ΔIDR-2	CGGAGCTCGAATTCGGATCCTTAATCTCCGGGCGCGGGGC
D/E-A-2	ACGGAGCTCGAATTCGGATCCTCAGCGGGCTGCGGCAGG
mGFP-DrHsp17-1	TACAAAGGTTCTGGTGGTTCTGGTATGTCGCAGAGCAACAA
Construction and complement of mutant stains	
Δdrhsp17-p1	CCTGACGAGCGGGAATACAAAAGAC
Δdrhsp17-p2	CCCAAGCTTGGCCGGAGGGTAGCAGGTCG
Δdrhsp17-p3	CGGGATCCTATGCATTCGGATTGAATCCAAAACA
Δdrhsp17-p4	GTCGGGGTCTTGCAGCTTCGT
Δdrhsp17-p5	AGAGCAACAACGAATCCGGCAA
Δdrhsp17-p6	GGTGGGACGCAGCTTCTCGA
P1-drhsp17-1	ATGCAGGAGGTACCAAGCACTAGTATGTCGCAGAGCAACAA
P1-drhsp17-2	CCTGCAGGTCGAATCGGATCCTCAGCGGTCTTCGTCAGGA
P1-ΔIDR-2	CCTGCAGGTCGAATCGGATCCTTAATCTCCGGGCGCGGGG
P1-D/E-A-2	CCTGCAGGTCGAATCGGATCCTCAGCGGGCTGCGGCAGG
P1-mGFP-drhsp17-1	GGTTCTGGTGGTTCTGGTATGTCGCAGAGCAACAACGAATCC
RT-qPCR primers	
qdrhsp17-1	CGAAGACGGCGGGCAACT
qdrhsp17-2	GGTGGGACGCAGCTTCTCG
qdr1343-1	GCCTACACCGAAGACCCCATTG
qdr1343-2	AAGAACTTGACGAGGTTGCCCAT
